# The potential impact of expanding target age groups for polio immunization campaigns

**DOI:** 10.1186/1471-2334-14-45

**Published:** 2014-01-29

**Authors:** Radboud J Duintjer Tebbens, Dominika A Kalkowska, Steven GF Wassilak, Mark A Pallansch, Stephen L Cochi, Kimberly M Thompson

**Affiliations:** 1 Kid Risk, Inc, 10524 Moss Park Road, Site 204-364, Orlando, FL 32832, USA; 2 Delft Institute of Applied Mathematics, Delft University of Technology, Delft, The Netherlands; 3 Global Immunization Division, Center for Global Health, Centers for Disease Control and Prevention, Atlanta, GA, USA; 4 Division of Viral Diseases, National Center for Immunization and Respiratory Diseases, Centers for Disease Control and Prevention, Atlanta, GA, USA; 5 University of Central Florida, College of Medicine, Orlando, FL, USA

**Keywords:** Polio, Eradication, Dynamic modeling, Disease outbreaks

## Abstract

**Background:**

Global efforts to eradicate wild polioviruses (WPVs) continue to face challenges due to uninterrupted endemic WPV transmission in three countries and importation-related outbreaks into previously polio-free countries. We explore the potential role of including older children and adults in supplemental immunization activities (SIAs) to more rapidly increase population immunity and prevent or stop transmission.

**Methods:**

We use a differential equation-based dynamic poliovirus transmission model to analyze the epidemiological impact and vaccine resource implications of expanding target age groups in SIAs. We explore the use of older age groups in SIAs for three situations: alternative responses to the 2010 outbreak in Tajikistan, retrospective examination of elimination in two high-risk states in northern India, and prospective and retrospective strategies to accelerate elimination in endemic northwestern Nigeria. Our model recognizes the ability of individuals with waned mucosal immunity (i.e., immunity from a historical live poliovirus infection) to become re-infected and contribute to transmission to a limited extent.

**Results:**

SIAs involving expanded age groups reduce overall caseloads, decrease transmission, and generally lead to a small reduction in the time to achieve WPV elimination. Analysis of preventive expanded age group SIAs in Tajikistan or prior to type-specific surges in incidence in high-risk areas of India and Nigeria showed the greatest potential benefits of expanded age groups. Analysis of expanded age group SIAs in outbreak situations or to accelerate the interruption of endemic transmission showed relatively less benefit, largely due to the circulation of WPV reaching individuals sooner or more effectively than the SIAs. The India and Nigeria results depend strongly on how well SIAs involving expanded age groups reach relatively isolated subpopulations that sustain clusters of susceptible children, which we assume play a key role in persistent endemic WPV transmission in these areas.

**Conclusions:**

This study suggests the need to carefully consider the epidemiological situation in the context of decisions to use expanded age group SIAs. Subpopulations of susceptible individuals may independently sustain transmission, which will reduce the overall benefits associated with using expanded age group SIAs to increase population immunity to a sufficiently high level to stop transmission and reduce the incidence of paralytic cases.

## Background

The global commitment to eradicate wild polioviruses (WPVs) and end poliomyelitis led to the launch of the Global Polio Eradication Initiative (GPEI) in 1988, which successfully reduced global polio incidence by 99% [1] and eradicated one of the three WPV serotypes (i.e., type 2 or WPV2) around 1999 [2]. By early 2014, all except three countries (i.e., Afghanistan, Nigeria and Pakistan) successfully interrupted indigenous transmission of WPV type 1 (WPV1) and no country has reported a case of WPV type 3 (WPV3) for over a year [3]. Despite these successes, the GPEI still needs to stop transmission of WPV1 everywhere contemporaneously and transition away from the use of oral poliovirus vaccine (OPV) to achieve the ultimate goal of ending all cases of poliomyelitis [4]. Partly due to importations into previously polio-free countries that caused outbreaks, the global annual incidence of paralytic polio remained around 1,000-2,000 cases between 2000 through 2010. The World Health Assembly in 2012 urged further intensification of the GPEI by declaring “the completion of global poliovirus eradication a programmatic emergency for global public health” ([5], p. 2). During 2012 the GPEI reported the fewest cases and smallest number of countries reporting cases in its history [6].

Successful polio eradication requires achieving and maintaining sustained high levels of population immunity until wild poliovirus transmission stops everywhere contemporaneously [4]. Although many high- and middle-income countries can maintain high population immunity by reaching and sustaining high routine immunization coverage, other countries must rely on the use of supplemental immunization activities (SIAs) that provide OPV during a short period of time regardless of immunization history to periodically and significantly boost population immunity [6]. Historically, SIAs emerged as a strategy to interrupt poliovirus transmission during the low season by “flooding the environment with vaccine virus so that a susceptible child has a lower probability of encountering wild virus and/or by rapidly increasing population immunity so that fewer susceptible children remain” ([7], p. 1332). The GPEI continues to rely heavily on SIAs to close immunity gaps resulting from sub-optimal routine immunization by targeting all children under 5 years of age [8]. Typically, polio SIAs focus on immunizing children younger than 5 years of age, because infants become susceptible once they lose protection from maternal antibodies and most people get exposed to circulating viruses during early childhood. Based on experience dating back to early OPV trials by Albert Sabin [9] and polio elimination from the Western Hemisphere [10-12], SIAs can dramatically reduce the prevalence of WPV and rapidly interrupt transmission. However, following the introduction of vaccination, the circulation of viruses may become more episodic, and those missed by vaccination may continue to remain susceptible and begin to accumulate.

With the passage of time since the interruption of WPV transmission in most places, importation outbreaks increasingly involve paralytic patients 5 or more years of age [13-17], which provides clear evidence that immunity gaps persist beyond early childhood in polio-free areas. Recognizing the need to protect older children and adults in these situations, the GPEI modified its outbreak response recommendations to explicitly expand the age group targeted in the first two SIAs up to 15 years of age or older depending on the context even in the absence of observed outbreak cases in older children and adults [8]. The failure of frequent SIAs targeting only children under 5 years of age to interrupt indigenous WPV transmission in the remaining endemic areas also raises the possibility that older children and adults may contribute significantly to the overall transmission of polioviruses in those places, even in the context of a substantial missed proportion of targeted children during SIAs. This would mean that expanding the target age groups for some SIAs in these areas may provide a strategy to more rapidly interrupt WPV transmission and maintain population immunity at a level high enough to prevent transmission. Alternatively, frequent SIAs targeting only young children may not prevent persistent transmission because they fail to protect enough children to completely stop transmission or they repeatedly miss relatively isolated subpopulations that sustain clusters of fully susceptible individuals. This would mean that reaching previously missed subpopulations may more rapidly interrupt transmission than expanding the target age range in the entire population.

Achieving WPV eradication is the primary objective of the GPEI. Further delay of the achievement of global WPV eradication and subsequent OPV cessation carries significant economic costs [18,19]. Donor fatigue and competition for public health resources during difficult economic times imply real financial risks for the GPEI. This paper evaluates the potential role and benefits of expanded age group vaccine campaigns using a differential-equation based poliovirus transmission model [20]. We explore different conditions and timing with respect to the implementation of expanded age group campaigns by considering several scenarios. We consider both preventive expanded age group SIAs occurring before outbreaks in previously polio-free areas or expanded age group SIAs prior to surges in incidence in endemic areas, and more reactive expanded age groups SIAs in the context of outbreak response in previously polio-free areas or efforts to accelerate the interruption of WPV transmission in endemic areas. Specifically, we model the potential impact of including older age groups in SIAs for three specific situations: alternative responses to the 2010 outbreak in Tajikistan, retrospective examination of elimination in northern India, and retrospective and prospective strategies to accelerate elimination in endemic northwestern Nigeria. While numerous inputs of the poliovirus transmission model remain highly uncertain [20-22], we assume for this analysis that the process of model validation across a wide range of situations produced representative generic model inputs that apply to poliovirus transmission in any situation (e.g., immunity states, waning process, OPV evolution process) as well as realistic characterization of the conditions in each of the three situations examined [20].

## Methods

We use a previously developed model of poliovirus transmission and consider three of the situations we used to fit generic model inputs [20]. Briefly, the deterministic, differential equation-based model includes immunity states to characterize the effect of recent (8 immunity states) or historic (24 immunity states) live poliovirus (LPV) infections and/or successful inactivated poliovirus vaccine (IPV) vaccinations. For this analysis, the 18 IPV-related immunity states remain inconsequential because we assume negligible IPV use in the three situations. Individuals in any active immunity state remain completely protected from paralysis if re-infected, but fully susceptible individuals and infants with insufficient maternal immunity (those who no longer possess protective levels of maternal antibodies) become subject to a serotype-specific, age-independent paralysis-to-infection ratio if infected [20]. The model characterizes the evolution process from OPV viruses towards WPV-like vaccine-derived polioviruses over 20 discrete stages, and explicitly considers serotype differences and both fecal-oral and oropharyngeal transmission [20].

The overall basic reproductive number (R_0_) characterizes the average number of secondary infections generated by combined fecal-oral and oropharyngeal transmission from one infected previously fully susceptible individual introduced in an entirely susceptible population. R_0_ for any population depends on hygienic and sanitary conditions, population density, mixing patterns, and climate, and may exhibit short-term oscillations due to seasonality as well as potential long-term trends. Given the different conditions that exist around the world, R_0_ varies between populations. The model assumes space-homogeneous mixing within each modeled (sub)population but includes age-heterogeneous mixing by assuming that individuals tend to mix preferentially with individuals of similar ages [20]. For each situation, we separately determined the proportion of contacts of any mixing age group (i.e., < 5, 5–14, or > 15 years of age) reserved for individuals of the same mixing age group (*κ*) to produce age distributions of cases consistent with the data. Specifically, the model assumes *κ* of 30%, 35%, and 40% of contacts in Tajikistan, northern India, and northwestern Nigeria, respectively, implying moderate preferential mixing within each mixing age group, with the remaining proportion of contacts occurring proportionately with individuals of all ages (i.e., including the mixing age group of the contacted individual) [20]. Aging represents a multi-stage process with transitions between any two age groups occurring according to a first-order, exponential expiry process [23,24], as specified in the model description [20]. Similarly, we characterize waning mucosal immunity, which influences the ability to participate in fecal-oral and oropharyngeal transmission, as a multi-stage process from high immunity to poliovirus transmission immediately after recovery from an LPV infection to reduced immunity to poliovirus transmission in the last waning stage [20]. We assume previously infected individuals reach the last waning stage after 4 years (Types 1 and 2) or 3 years (Type 3) on average in the absence of further infections [20]. Additional file 1 provides the equations for the force-of-infection in the model with age-mixing and multiple subpopulations as well as the waning curves.

To characterize the stochastic event of die-out (elimination) of transmission of each LPV virus (i.e., WPV or OPV-related virus in each reversion stage) in a population in our deterministic model, we fixed a transmission threshold of the effective infectious proportion (i.e., the number of infectious people in a population, weighted by their relative infectiousness to others, divided by the total number of people in the same population) below which the force-of-infection of a LPV becomes 0 in the model [20]. We define the threshold as a proportion rather than an absolute number to avoid model artifacts related to population size. Specifically, using a threshold of 1 infection in a large population would eventually lead to circulating vaccine-derived polioviruses (cVDPVs) following a SIA round in a WPV-free setting with low or no routine immunization coverage, because it would take a long time for the prevalence in the last reversion stage to drop below 1, while cVDPVs would not emerge in a very small population in which the prevalence would never exceed 1 in higher reversion stages. We apply the threshold for each mixing age group and subpopulation separately, which means that virus can continue to circulate in one of these groups while giving a force-of-infection of 0 to other groups. For mixing between sub-populations we assume highly preferential mixing so that the time of elimination can differ between subpopulations substantially (especially if their immunity levels differ significantly). For mixing between age groups we assume relatively much less preferential mixing, so that elimination times between age groups remain very similar. This approach provides a very crude method to approximate die-out that occurs in actual populations and depends on local heterogeneity and chance. However, the threshold we determined generally produced elimination times consistent with the evidence [20] and allows us to explore the relative differences in potential elimination times between scenarios, even if the actual elimination times in the model do not reflect the stochasticity of actual virus elimination.

We conducted extensive literature reviews with experts in the field [21,25] and elicited numerical values from experts to estimate initial ranges of values for model inputs related to poliovirus transmission and immunity [22]. We then calibrated the model within the ranges obtained from the expert review process by visually fitting historical poliomyelitis incidence data and other features of poliovirus transmission in 9 different situations, including the emergence of cVDPVs and WPV elimination times, the age distribution of cases, and estimates of zero-dose children derived from surveillance data (see Additional file 1). We adopt the resulting generic and situation-specific model inputs for this analysis [20] (specifically, Tables I, VII, XI, and XII for generic, Tajikistan, northwestern Nigeria, and northern India inputs, respectively in the prior publication). However, we modified the characterization of SIAs for prospective use of the model to allow it to directly specify the true coverage of individual rounds and the probability of children repeatedly receiving or missing doses (see Additional file 1). Briefly, the approach requires specifying the true coverage (*TC*) of each SIA, defined as the fraction of the targeted population receiving a dose, and the repeated missed probability (*P*_*RM*_), defined as the conditional probability that a targeted individual does not receive a dose in a round, given that the individual did not receive a dose in the previous round despite falling into the targeted population for that round. From these, we calculate the repeated reached probability (*P*_*RR*_), defined as the conditional probability that a targeted individual receives a dose in a round, given that the individual received a dose in the previous round. In the model, we then track which of the fully susceptible and maternally immune individuals (i.e., defined as children born with maternal antibodies transferred to them by an immune mother) at the time of an SIA are: (1) children born since the last SIA, subject to a coverage of *TC*, (2) children who did not receive a dose in the previous round, subject to a coverage of 1-*P*_*RM*_, and (3) children who received a dose in the previous SIA but remain fully susceptible or maternally immune due to failure of the vaccine to take, subject to a coverage of *P*_*RR*_. To ensure that the overall coverage matches the specified *TC* in each round, we evenly divide all remaining doses over all targeted individuals with active immunity (i.e., individuals not in the fully susceptible or maternally immune state). Fractional rounds target less than the whole modeled population target age range (e.g., only one district when the modeled population represents a state) and reduce each coverage by the same fraction targeted (*F*). For the under-vaccinated subpopulations that we characterize in northern India and northwestern Nigeria, we specify a relative coverage for this subpopulation compared to the general population (see Additional file 1).

For all three situations, we compared the starting point for the model to the best information available to us as of August 2013. For all analyses, we keep all model inputs constant except for those vaccination inputs that specify the different policy scenarios (e.g. target age groups of an SIA). For Tajikistan, we modeled a single population, while for northern India and northwestern Nigeria we modeled 90% of the population as the general population and 10% as a relatively isolated subpopulation. Following the publication of our earlier analysis [20], more complete data became available for analysis on the cases that occurred in the Tajikistan outbreak and the dose histories of all acute-flaccid paralysis (AFP) cases since 1997 for northern India and since 2003 for northwestern Nigeria. We also updated the original demographic data [26] with the 2012 revision from the United Nation’s Population Division World Population Prospects [27]. These data allowed us to better assess the age distribution of cases and outbreak kinetics for Tajikistan and the historic proportions of missed children for northern India and northwestern Nigeria. Applying the new characterization of SIAs to ensure consistency with the historic reported zero-dose proportions among non-polio AFP (NPAFP) cases over time, rather than estimates based on expert judgment, led to some adjustments of generic and situation-specific model inputs in order to produce more realistic historic poliomyelitis incidence (see Additional file 1, Table 1).

**Table 1:** **Model inputs that differ from those developed previously**[20]**after consideration of additional situation-specific data and revised SIA characterization**

**Model input (symbol)**	**Value in Duintjer Tebbens et al. (2013) [**[20]**]**	**New value**
**Generic model inputs:**		
Ratio of R_0_ for PV3 vs. PV1	0.8	0.75
Average time to reach last reversion stag (ϵ, in days) (for PV1; PV2; PV3)^a^	547.5; 360; 547.5	620.5; 408; 620.5
Basis for demographic data	World Population Prospects: The 2010 Revision [26]	World Population Prospects: The 2012 Revision [27]
**Tajikistan model inputs:**		
Relative routine immunization coverage compared to most recent survey (used from 1992 forward)	0.9	0.88
**Northern India model inputs:**		
Number of subpopulations (Bihar)	1	2
Size of subpopulation relative to total population		
- Bihar	NA	10%
- Western Uttar Pradesh	4%	10%
Proportional change in R_0_ due to seasonality (α)	0.25	0.20
Peak day of seasonal amplitude, WUP	195 (July 14)	165 (June 14)
Proportion of potentially infectious contacts of individuals in the under-vaccinated subpopulation with other individuals in the under-vaccinated subpopulation (*p*_*within*_)		
- Bihar	NA	0.70
- Western Uttar Pradesh	0.95	0.70
Routine immunization coverage in the under-vaccinated subpopulation	Increasing over time	0
Per-dose take rate (tr) (PV1; PV2; PV3)		
- tOPV	0.35; 0.50; 0.30	0.35; 0.60; 0.27
- mOPV	0.50; NA; 0.45	0.45; NA; 0.45
- bOPV	0.45; NA; 0.40	0.42; NA; 0.42
**Northwestern Nigeria model inputs:**		
R_0_	8.0	7.5
Proportional change in R_0_ due to seasonality (α)	0.05	0.10
Day of seasonal peak in R_0_ (*pd*)	60 (March 1)	100 (April 10)
Basis for routine immunization coverage^b^	POL coverage	DTP coverage
Routine immunization coverage relative to reported data		
- General population	1.08	1
- Under-vaccinated subpopulation	0.29	0
Per-dose take rate (*tr*) (PV1; PV2; PV3)		
- tOPV	0.45; 0.7; 0.4	0.45; 0.7; 0.35
- mOPV	0.66; NA; 0.65	0.6; NA; 0.6
- bOPV	0.54; NA; 0.52	0.54; NA; 0.54

^a^The original and new values equal 1.5 and 1.7 times the estimated time to reach viral protein 1 divergence thresholds defined by the Global Polio Laboratory Network, [28,29], respectively.

^b^Continued discussion of available routine immunization data in Nigeria determined that DTP coverage probably provides a more reliable estimate of routine immunization coverage for polio vaccine doses, because reported polio vaccine doses likely includes doses administered as part of SIAs.

Table 1 summarizes the inputs that we updated based on improved information and better characterization of SIAs, with all other input values in the model remaining the same as reported earlier (see Additional file 1) [20]. In the context of implementing the refined approach to SIA characterization, we assume the same true coverage by subpopulation for the expanded age group SIAs as for children under 5 years of age (i.e., expanded age groups SIAs imply the same coverage applied to a larger target age range and the corresponding increase in vaccine doses used). The assumption about the repeated missed probabilities for older children and adults results in negligible impact because a high proportion of individuals in the expanded age groups benefit from actively acquired immunity, while we distinguish repeated missed and repeated reached probabilities only for fully susceptible and maternally immune individuals.

We compute the number of doses administered for any given SIA as the sum over each population of *F* × *TC* × *N*_*target*_, where the size of the target population *N*_*target*_ comes directly from the model. The number of doses distributed differs from the doses administered, depending on the wastage for the modeled situations. While we considered data from the GPEI on doses planned nationally for each SIA, we do not know how many doses the modeled states actually administered, so we estimated these in the model.

### Tajikistan

The column on the left of Table 2 lists the scenarios we considered for Tajikistan. The model considers the 3 administrative regions primarily affected by the 2010 outbreak (i.e., approximately two-thirds of the total Tajikistan population) as one homogeneously mixing population [20]. The model focuses on the large WPV1 importation outbreak in 2010 that occurred in the context of sub-optimal routine vaccination coverage since the last SIAs in the early 2000s [14]. The reference case represents the actual response to the 2010 outbreak that occurred, using the reported target ages for each outbreak response SIA (oSIA) round of 0–5 years for the first two rounds and 0–14 years for the 4 subsequent rounds [20]. For the alternative scenarios, we considered the possibility that all oSIA rounds targeted only 0–5 year olds, or that they all targeted 0–14 year olds, and we also considered the size of the outbreak in the modeled (closed) population of the 3 affected regions if no response occurred. Given that the outbreak probably reached its peak in this population by the time the first large oSIA round occurred, we also considered the impact of the same response scenarios, but with each oSIA round conducted 30 days earlier. Finally, we considered the impact of one or two hypothetical preventive SIAs (pSIAs) in 2009 targeting 0–5 or 0–14 year olds and attaining 80% true coverage per round, which the country considered but did not conduct due to insufficient funds [4]. Given that even a single pSIA targeting 0–5 year olds prevents the outbreak altogether in the model with all else equal, we constructed a hypothetical reference case scenario as a comparator for the pSIAs. After observing that the proposed 2009 pSIA would prevent the 2010 outbreak entirely in the model, we explored the impact of a potential WPV1 introduction on a different dates to assess the robustness of the results. We found that if we introduce WPV1 one month later (i.e., 12/1/2009 instead of 11/1/2009) the importation will lead to transmission even with the 2009 pSIA, because of the higher seasonal R_0_ at the time of introduction. For this hypothetical alternative scenario with importation on December 1, 2009, the cumulative incidence reaches one paralytic case 7 days later than for the reference case with November 1 WPV introduction, and thus we also move all oSIA rounds by 7 days for the hypothetical alternative case comparator, which we use to further explore the impact of different pSIA scenarios. Similarly, for each of the four pSIA scenarios, we keep the time between the incidence reaching one cumulative paralytic case and the oSIAs constant. We assume that the generally much smaller outbreaks that occur after the pSIAs trigger two oSIA rounds that target 0–5 year olds (Table 2).

**Table 2:** **Details of scenarios and estimates of the number of paralytic cases prevented, reduction of time until WPV elimination by serotype, and vaccine doses administered compared to the reference case for Tajikistan with WPV1 imported on November 1, 2009 (top) and the hypothetical alternative reference case with WPV1 imported on December 1, 2009 (bottom) (see Additional file****1****and Duintjer Tebbens et al. (2013)**[20]**for other model assumptions fixed across all scenarios)**

**Expanded age group scenario**	**Target age groups of 2010 oSIAs (in years), by round**	**Dates (vaccines) of 2010 oSIAs, by round**	**Target age groups of 2009 pSIA(s)**	**Dates (vaccines) of 2009 pSIA(s) (in years), by round**	**Paralytic cases prevented, 2010 [%]**	**Reduction in WPV1 re-interruption time (days)**	**Additional vaccine doses administered (millions)**
Reference case: Actual target ages	0-5 (first 2) then 0–14 (last 4)	5/4 (mOPV1) 5/18 (mOPV1) 6/1 (mOPV1) 6/15 (mOPV1) 10/4 (tOPV) 11/8 (tOPV)	None		Reference	Reference	Reference
No response	None		None		-195 [-40%]	-55	-7.7
All oSIAs target 0–5 year olds	0-5 (all rounds)	5/4 (mOPV1) 5/18 (mOPV1) 6/1 (mOPV1)	None		-2 [0%]	-6	-3.7
All oSIAs target 0–14 year olds	0-14 (all rounds)	6/15 (mOPV1) 10/4 (tOPV) 11/8 (tOPV)	None		19 [4%]	3	1.9
Actual target ages, start each round 30 days earlier	0-5 (first 2) then 0–14 (last 4)	4/4 (mOPV1) 4/18 (mOPV1)	None		291 [61%]	23	0.0
All oSIAs target 0–5 year olds, start each round 30 days earlier	0-5 (all rounds)	5/2 (mOPV1) 5/16 (mOPV1)	None		287 [60%]	10	-3.7
All oSIAs target 0–14 year olds, start each round 30 days earlier	0-14 (all rounds)	9/4 (tOPV) 10/8 (tOPV)	None		312 [65%]	29	1.9
One pSIA in Spring 2009 targeting 0–5 year olds	None		0-5	5/4 (tOPV)	481 [100%]	243	-7.1
Hypothetical alternative reference case: Actual target ages, WPV1 introduction 30 days later, start each oSIA 7 days later	0-5 (first 2) then 0–14 (last 4)	5/11 (mOPV1) 5/25 (mOPV1) 6/8 (mOPV1) 6/23 (mOPV1) 10/11 (tOPV) 11/15 (tOPV)	None		Alternative reference	Alternative reference	Alternative reference
One pSIA targets 0–5 year olds in Spring 2009, two oSIAs target 0–5 year olds	0-5 (both rounds)	6/7 (mOPV1) 6/21 (mOPV1)	0-5	5/4 (tOPV)	430 [89%]	-9	-5.8
One pSIA targets 0–14 year olds in Spring 2009, two oSIAs target 0–5 year olds	0-5 (both rounds)	6/15 (mOPV1) 6/29 (mOPV1)	0-14	5/4 (tOPV)	450 [94%]	-6	-5.3
Two pSIAs target 0–5 year olds in Spring 2009, two oSIAs target 0–5 year olds	0-5 (both rounds)	7/7 (mOPV1) 7/21 (mOPV1)	0-5 (both rounds)	5/4 (tOPV) 6/4 (tOPV)	471 [98%]	0	-5.0
Two pSIAs target 0–14 year olds in Spring 2009, two oSIAs target 0–5 year olds	0-5 (both rounds)	8/3 (mOPV1) 8/17 (mOPV1)	0-14 (both rounds)	5/4 (tOPV) 6/4 (tOPV)	476 [99%]	3	-3.7

### Northern India

The column on the left of Table 3 lists the scenarios we considered for northern India. The model separately considers endemic WPV transmission in the entire state of Bihar and 25 districts comprising the last reservoir of indigenous poliovirus transmission in Western Uttar Pradesh (WUP) [20]. Unlike the previous fit based on more limited data about missed children that modeled Bihar as one homogeneous population [20], we assume both Bihar and WUP include relatively large preferentially mixing subpopulations (i.e., 10% of the total population in each area) with sub-optimal, but progressively improving SIA coverage. The model focuses on reproducing the pattern of endemic circulation and elimination of all three WPVs as well as an outbreak following a reintroduction of WPV3 from WUP into Bihar in 2007 and the small type 2 cVDPV outbreak in WUP in 2009–2010 (see Additional file 1 for more details).

**Table 3:** **Details of scenarios and estimates of the number of paralytic cases prevented, reduction of time until WPV elimination by serotype, and vaccine doses administered compared to the reference case for northern India (see Additional file****1****and Duintjer Tebbens et al. (2013)**[20]**for other model assumptions fixed across all scenarios)**

**Expanded age group scenario**	**Target age groups of 2008 SIAs (in years), by round**	**Paralytic cases prevented, 2008–2012 (WPV1&3) [%]**	**Reduction in WPV elimination time (days) **			**Additional vaccine doses administered (millions)**	
				**PV1**	**PV3**		**PV1 & 3**
**Bihar**^**a**^							
Reference case: No expanded age groups	0-4 (all rounds)	Reference	Reference	Reference	Reference	Reference	
Expand Jan 2008 (tOPV) SIA through 14 year olds	0-14 (Jan); 0–4 (all other rounds)	45 [11%]	35	1	1	16.0	
Expand Jan 2008 (mOPV1) SIA through 14 year olds	0-14 (Jan); 0–4 (all other rounds)	26 [6%]	43	0	0	15.9	
Expand Jun 2008 (mOPV3) SIA through 14 year olds	0-14 (Jun); 0–4 (all other rounds)	25 [6%]	0	5	5	15.7	
Expand Jul 2008 (mOPV1) SIA through 14 year olds	0-14 (Jul); 0–4 (all other rounds)	19 [5%]	49	0	0	16.1	
Coverage in under-vaccinated subpopulation increased by 0.2 during Jun (mOPV3) SIA	0-4 (all rounds)		47 [12%]	0	9	9	0.2
Expand Jun (mOPV3) SIA to all ages with coverage in under-vaccinated subpopulation increased by 0.2	All ages (Jun); 0–4 (all other rounds)	97 [24%]	0	152	152	71.7	
Coverage in under-vaccinated subpopulation increased by 0.2 during Jul (mOPV1) SIA	0-4 (all rounds)	16 [4%]	26	0	0	0.2	
Expand Jul (mOPV1) SIA to all ages with coverage in under-vaccinated subpopulation increased by 0.2	All ages (Jul); 0–4 (all other rounds)	43 [11%]	417	0	0	73.1	
**Western Uttar Pradesh**^**b**^							
Reference case: No expanded age groups	0-4 (all rounds)	Reference	Reference	Reference	Reference	Reference	
Expand Jan 2008 (tOPV) SIA through 14 year olds	0-14 (Jan); 0–4 (all other rounds)	34 [6%]	28	2	2	10.5	
Expand Jan 2008 (mOPV1) SIA through 14 year olds	0-14 (Jan); 0–4 (all other rounds)	24 [5%]	33	0	0	10.4	
Expand Jun 2008 (mOPV3) SIA through 14 year olds	0-14 (Jun); 0–4 (all other rounds)	26 [5%]	0	-1	-1	10.3	
Expand Jul 2008 (mOPV1) SIA through 14 year olds	0-14 (Jul); 0–4 (all other rounds)	28 [5%]	40	0	0	10.4	
Coverage in under-vaccinated subpopulation increased by 0.2 during Jun (mOPV3) SIA	0-4 (all rounds)		39 [7%]	0	-11	-11	0.1
Expand Jun (mOPV3) SIA to all ages with coverage in under-vaccinated subpopulation increased by 0.2	All ages (Jun); 0–4 (all other rounds)	143 [27%]	0	-33	-33	47.6	
Coverage in under-vaccinated subpopulation increased by 0.2 during Jul (mOPV1) SIA	0-4 (all rounds)	23 [4%]	31	0	0	0.1	
Expand Jul (mOPV1) SIA to all ages with coverage in under-vaccinated subpopulation increased by 0.2	All ages (Jul); 0–4 (all other rounds)	55 [10%]	299	0	0	47.7	

^a^1/6 (tOPV and mOPV1) 6/1 (mOPV3) 7/6 (mOPV1).

^b^1/6 (tOPV and mOPV1) 6/8 (mOPV3) 7/6 (mOPV1).

We explore several hypothetical retrospective scenarios during the year 2008, when the Government of India significantly intensified its immunization of children under 5 years of age. The reference case represents the calibrated model involving only SIAs targeting 0–4 year olds. For the expanded age group scenarios, we remain limited by the actual SIAs that occurred during 2008, which includes fractional and full type 1 monovalent OPV (mOPV1) and type 3 monovalent OPV (mOPV3) rounds and two mixed mOPV1/tOPV SIAs in early 2008. Table 3 shows the expanded age group scenarios we chose based on the available rounds. The first four scenarios represent arguably realistic expanded age group scenarios involving a single round expanded to 0–14 year olds, with different vaccines (tOPV, mOPV1, or mOPV3) and two different timings for the mOPV1 round (i.e., during low or high season). All of the rounds assume low true SIA coverage of between 0.05 and 0.25 for the under-vaccinated subpopulation for the reference case, which we recognize limits the potential benefit of the expanded age group scenario. Therefore, we also explored four scenarios involving an increase by 0.2 of the SIA coverage in the under-vaccinated subpopulation during the high season mOPV1 or mOPV3 round, with or without expanding the target age range to all ages in order to show the maximum possible benefit of expanded SIAs (Table 3).

### Northwestern Nigeria

The column on the left of Table 4 lists the scenarios we considered for northwestern Nigeria. The model considers the 7 states that comprise the Northwest zone of the country, representing approximately a quarter of the total Nigerian population [20]. As in Bihar and WUP, we assume that approximately 10% of the Northwest zone remains chronically under-vaccinated and preferentially mixes with itself to sustain indigenous WPV transmission even when the general population became better-immunized [20]. The model focuses on reproducing the pattern of endemic circulation and elimination of all three WPVs as well as the large and prolonged type 2 cVDPV outbreak that started around 2005 [30] (see Additional file 1). The reference case represents continuation of the *status quo* from 2013 forward, assuming continued true coverage of 0.85, repeated missed probability of 0.85, and relative coverage of 0.2 in the subpopulation at the level reached in late-2013 in the retrospective model. The planned SIA calendar for 2013 includes SIAs targeting all of the Northwest zone in each month except August, with tOPV in March and September and type 1 and 3 bivalent OPV (bOPV) in all other months. For subsequent years, we assume that SIAs with those vaccines continue to occur on the 15^th^ of each month except in August in the northwest. We focus on the results for WPV1 because the model eliminated WPV3 by early-2013 and we do not explicitly include any re-importation while type 2 cVDPVs continue at a very low level, although we did not find that they disappear completely based on our assumed projected tOPV vaccination intensity (see Additional file 1).

**Table 4:** **Details of scenarios and estimates of the incremental number of paralytic cases prevented, reduction of time until WPV elimination by serotype, and vaccine doses needed compared to the reference for northwestern Nigeria (see Additional file****1****and Duintjer Tebbens et al. (2013)**[20]**for other model assumptions fixed across all scenarios)**

**Expanded age group scenario**	**Target age groups of SIAs (in years), by round**^**a**^	**Paralytic cases prevented (2012–2015) [%]**	**Reduction in WPV1 elimination time (days)**	**Additional vaccine doses administered (through 2014) (millions)**
Reference case: No expanded age groups	0-4 (all rounds)	Reference	Reference	Reference
**Hypothetical retrospective scenarios:**				
Relative coverage in under-vaccinated during Feb (bOPV) and Mar (tOPV) 2012 SIAs increased by 0.2	0-4 (all rounds)	93 [53%]	56	0.2
Expand Feb (bOPV) and Mar (tOPV) 2012 SIAs to all ages	All ages (Feb and Mar 2012); 0–4 (all other rounds)	76 [44%]	49	46.3
		11/10/2012 (bOPV)		
Expand Feb (bOPV) and Mar (tOPV) 2012 SIAs to all ages with relative coverage in under-vaccinated subpopulation increased by 0.2	All ages (Feb and Mar 2012); 0–4 (all other rounds)	130 [75%]	302	46.5
**Prospective scenarios:**				
Expand Nov 2013 bOPV SIAs through 14 year olds	0-14 (Nov 2013); 0–4 (all other rounds)	1 [0.6%]	17	9.3
Expand Nov 2013 bOPV SIAs to all ages	All ages (Nov 2013); 0–4 (all other rounds)	2 [1.0%]	32	29.6
Relative SIA coverage in under-vaccinated subpopulation increased by 0.05 from Nov 2013 on	0-4 (all rounds)	2 [1.2%]	58	0.4

Notes:

^a^SIAS in 2012 on 2/18 (bOPV), 3/31 (tOPV), 5/12 (bOPV), 7/7 (bOPV), 9/15 (bOPV), 10/4 (bOPV), 11/10 (bOPV), 12/1 (tOPV), and 12/15 (bOPV). SIAs in 2013 on 1/12 (bOPV and tOPV), 2/2 (bOPV), 3/2 (tOPV), 4/20 (bOPV), 5/11 (bOPV), 6/15 (bOPV), 7/6 (bOPV), 9/7 (tOPV), 10/12 (bOPV), 11/9 (bOPV), 12/14 (bOPV). For 2014 and 2015, we assume SIAs occur on the 15th of all months except August.

The expanded age group scenarios we consider include two prospective scenarios that expand the November 2013 SIA with bOPV either up through 14 year olds or to all ages. For comparison, we explore a scenario of increasing the relative SIA coverage in the under-vaccinated subpopulation compared to the general population by 0.05 from November 2013 through the end of 2014. The reference case in the model already represents an apparent path to WPV1 elimination assuming a sustained commitment to the current SIA schedule (with relatively few cases expected in the model after November 2013). Low coverage in the under-vaccinated subpopulation may limit the benefit of expanded age group campaigns. Consequently, we explore a number of hypothetical retrospective scenarios during 2012 to consider the maximum potential benefit of SIAs with higher coverage in the under-vaccinated subpopulation and at a time when a resurgence of WPV1 occurred. Specifically, we considered two scenarios that assume 0.2 higher relative SIA coverage in the under-vaccinated subpopulation compared to the general population during the February and March 2012 rounds. In one scenario, we assume these rounds target only 0–4 year olds (as happened in reality), while in the other we expanded the target age range to include all ages. We also considered a scenario of expanding SIAs to all ages without the improvement in the relative coverage in the under-vaccinated subpopulation (Table 4).

## Results

### Tajikistan

**Figure 1:**
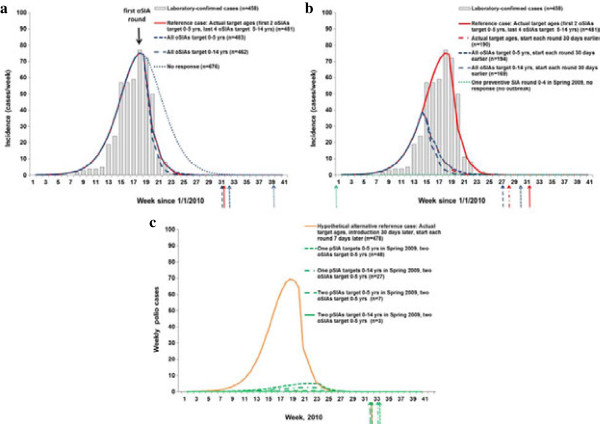
**The impact of expanded age groups and other scenarios on incidence for the 2010 Tajikistan WPV1 outbreak.** Arrows indicate the time of elimination in the model for each scenario. **(a)** Actual dates of outbreak response (oSIAs). **(b)** Impact of earlier oSIA rounds and preventive SIA rounds (pSIAs) in Spring 2009. **(c)** Hypothetical alternative outbreak assuming WPV1 introduction 30 days later.

Figure 1a shows the results of varying the target age groups for the oSIAs conducted after detection of the 2010 WPV1 outbreak in Tajikistan. For this closed population representing the three administrative regions affected by the outbreak [20], the model fit for the reference case appears broadly consistent with the data. The model estimates a total of 481 cases in 2010, including oSIAs that required an estimated 7.7 million doses (without consideration of wastage), and re-interruption of WPV1 occurring in August 2010. The outbreak appears to reach its peak in this simulation due to natural burnout before the first oSIA round, as demonstrated by the “No response” curve. Consequently, the oSIAs only shift the incidence curves slightly to the left after they occur, although they lead to a significant reduction of 195 estimated cases (see Table 2) and an expected reduction of 55 days in the time to re-interrupt transmission compared to no response. The outbreak response also probably prevented exportation and more extensive spread to other populations than the 18 linked cases reported from three neighboring countries [14], although it occurred too late to prevent these exportations. Varying the target age groups similarly produces relatively little impact on the overall transmission dynamics, but we expect 2 additional cases if all four oSIAs targeted 0–5 year olds and a total of 19 fewer cases if all oSIAs targeted 0–14 year olds. The last column in Table 2 shows the additional doses implied by the scenario. Given our assumption of a constant case-fatality rate by age for poliomyelitis, which some evidence suggests may underestimate the number of fatalities and severity of paralysis in older children and adults [31], we may slightly underestimate both the expected burden of disease and the clinical benefit of targeting older age groups in oSIAs. We do not anticipate a large difference in the total numbers of cases, but we recognize the high visibility of any deaths.

Figure 1b shows that conducting the oSIAs 30 days earlier prevents more than half of the cases in this population (i.e., 287 or more of 481 cases, depending on the target age groups), without costing any more doses. This result reaffirms the analysis of outbreak responses that “faster is better” [32]. However, the difference between various choices of target age groups remains relatively small even with an earlier response, with 25 additional cases prevented if the first two oSIA rounds target all children under 15 years of age instead of only children under 6 years of age. More importantly, in the context of managing population immunity that focuses on prevention [4], Figure 1b shows that with all else equal, a single pSIA round, as considered in 2009 [4], prevents the outbreak following the WPV1 importation from India [14] into Tajikistan, because it raises population immunity high enough over the threshold to prevent substantial transmission.

We recognized the fragility of this finding to the assumed but uncertain November 1, 2009 date of WPV1 importation, due to seasonal fluctuation in R_0_ and population immunity remaining near the threshold even with the pSIA. We used the model to explore the impact of different WPV1 importation dates and found that by moving the WPV1 introduction date to December 1 (i.e., 30 days later) we observe a small outbreak even with a pSIA. We model this alternative importation using the scenario identified at the bottom of Table 2 as the hypothetical alternative reference case. Figure 1c shows the results of the hypothetical alternative reference case and four preventive SIA scenarios, with the oSIAs timed to occur at the same time relative to when the cumulative incidence exceeds 1 paralytic case (i.e., assuming the same time between the actual oSIAs in Figure 1a and the response-triggering signal of 1 cumulative paralytic case in the model). The hypothetical alternative reference case with response (Figure 1c) leads to an almost identical outbreak curve compared to the reference case with response (Figure 1a) with slightly fewer total cases (case estimates indicated in the legends following the scenario names). We find that even with only two oSIAs in 2010, conducting preventive campaigns in 2009 reduces the size of the outbreak that would have resulted from a later WPV1 importation, which decreases the total number of doses (and vaccine costs) significantly. All four pSIA scenarios prevent the vast majority of cases and lead to significant savings in numbers of doses administered compared to the hypothetical reference case. The pSIA scenarios listed in Table 2 do not significantly affect the time of die-out. Overall, for pSIAs, we find a significant reduction of approximately 50% of the number of cases between targeting 0–5 and 0–14 year olds (Figure 1c). This finding remains consistent with the existence of a sizeable number of fully susceptible people in the age group 6–14 years old, as reflected in the reported age distributions of cases [14]. It also shows that immunizing some of these susceptible individuals as well as boosting individuals with waned immunity in this age group provides sufficient added population immunity to poliovirus transmission to significantly influence the outbreak size and dynamics if any importation occurs the following year. However, Figure 1c also shows that adding a second pSIA targeted at 0–5 year olds prevents more cases than expanding a single pSIA through 14 years of age because of a combination of poor tOPV take (i.e., 40% for type 1) and suboptimal coverage of each pSIA (i.e., 80%).

### Northern India

**Figure 2:**
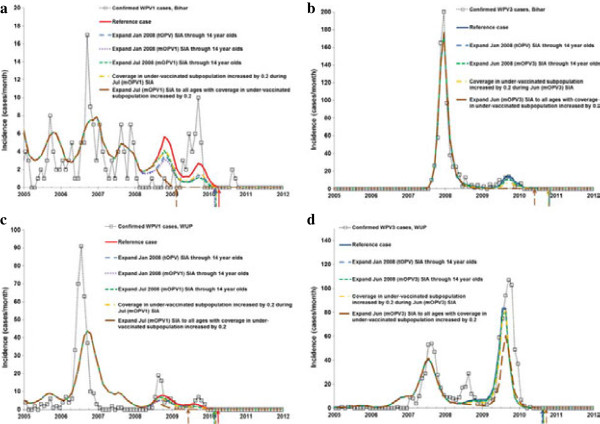
**The impact of expanded age groups and other scenarios on WPV1 incidence in northern India.** Arrows indicate the time of elimination in the model for each scenario. **(a)** Bihar – type 1, **(b)** Bihar – type 3, **(c)** Western Uttar Pradesh (WUP) – type 1, **(d)** WUP – type 3.

The model for the reference case for Bihar estimates 410 cases from 2008–2012, elimination of WPV1 in April 2010 and WPV3 in October 2010, and 70.2 million vaccine doses administered during 2008. The model for the reference case for WUP estimates 532 cases during 2008–2012, elimination of WPV1 in April 2010 and WPV3 in August 2010, and 50.5 million vaccine doses administered during 2008. Figure 2 shows how the expanded age group and other scenarios change the time series of incidence of paralytic cases due to WPV1 and WPV3 in northern India. While the two states and WPV serotypes in Figure 2 involve dramatically different incidence patterns, the qualitative impact of the expanded age groups remains comparable. Conducting a single expanded age group round targeting 0–14 years with no improvement in coverage for the under-vaccinated sub-population remains roughly equivalent to an increase of 0.20 in the coverage for the under-vaccinated sub-population without expanded age groups. The difference between conducting a mOPV1 expanded age group SIA in the low or high season remains relatively small. For both serotypes, the expanded age group SIAs with mOPV increase population immunity more than SIAs with tOPV, although using tOPV may eliminate both types earlier with a single expanded age group SIA round. A substantial reduction in the elimination time occurs for WPV3 in the scenarios we explored for Bihar (top of Table 3) but not for WUP (bottom of Table 3) even in the case of SIAs expanded to all ages. The lack of a reduction in the WPV elimination date relates to the timing of the expanded age group SIA relative to the natural transmission dynamics (Table 3 and Figure 2). Most notably, for WPV3 in WUP, we see that expanded age group SIAs in 2008 reduce the large peak in WPV3 incidence in 2009. This translates to later elimination in the model because a higher WPV3 peak in 2009 following the outbreak helps with die-out in the model due to the relatively larger increases in population immunity above the threshold needed to push the WPV3 prevalence to elimination. The maximum benefit (reduced cases and elimination time) of an expanded age group SIA in northern India occurs if coupled with an increase in coverage in the under-vaccinated subpopulation and if it includes all ages (Figure 2). Comparison to the scenario with only increased coverage in the under-vaccinated subpopulation but without expanded age groups reveals a significant effect of the expanded age group strategy. Addition of a mOPV SIA targeted at all ages leads to considerably more prevented cases and may shift the modeled elimination time of the targeted serotype by over a year (i.e., WPV1 in Bihar in the top of Table 3). This comes at a cost of almost 120 million additional doses administered in the two states, but it may potentially save vaccine doses overall by allowing for earlier reduction in the use of the monovalent vaccine.

### Northwestern Nigeria

**Figure 3:**
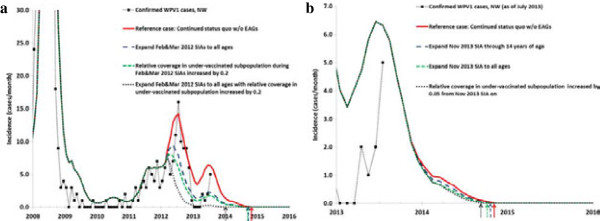
**WPV1 incidence for expanded age group and other scenarios in northwestern Nigeria.** Arrows indicate the time of elimination in the model for each scenario. **(a)** Hypothetical retrospective policy changes starting in 2012, **(b)** Prospective policy changes starting late 2013 or later.

The reference case leads to an estimated 174 WPV1 cases during 2012–2015, elimination of WPV type 1 in November 2014, and almost 190 million vaccine doses administered during 2012–2014. Figure 3 shows how the expanded age group SIAs and other scenarios reduce the incidence of paralytic cases due to WPV1 in northwestern Nigeria. The model suggests that with consistent true coverage and repeated missed probabilities for SIAs going forward in both the general and under-vaccinated subpopulations, WPV1 elimination will occur in the second half of 2014 in the absence of any expanded age group SIAs. If the projected rounds do not occur or do not sustain high coverage in the general population while also consistently reaching the under-vaccinated subpopulation, then we could see a different path with a resurgence in cases.

Figure 3a considers the impact of hypothetical alternative strategies retroactively applied from 2012, which show missed opportunities to prevent cases and reduce elimination time. If northern Nigeria had conducted two consecutive rounds in early 2012 and reached the under-vaccinated subpopulation much better than we assumed for the reference case (i.e., relative SIA coverage increased by 0.20 from 0.25), then this prevents the entire resurgence of cases in 2012 even without expanded age groups. This scenario reduces the WPV1 elimination time in the model by almost 2 months (Table 4). The expanded age group scenario in Figure 3a shows the expected benefit of vaccinating all ages if these rounds attain the same improved coverage in the under-vaccinated subpopulation, with a shift in WPV1 elimination time of almost 1 year (Table 4). This scenario involves almost 50 million additional doses administered, which does not account for any fewer doses needed after the achievement of elimination. Notably, real shortages in vaccine supplies limited the willingness to consider expanded age group SIAs in 2012. The difference between these two scenarios suggest that expanding the age groups of the SIAs may under certain circumstances provide the additional jump in population immunity necessary to interrupt transmission, even if the majority of fully susceptible people remain younger than 5 years of age. Assuming that relatively large groups of preferentially mixing under-vaccinated people in an otherwise well-vaccinated general population of Nigeria represent the main drivers of sustained WPV1 circulation, then the expanded age group scenario in Figure 3a represents an upper bound for the potential benefits (prevented cases and elimination time) of using expanded age groups in northwestern Nigeria, with two rounds targeting all ages coupled with successful efforts to reach previously missed populations for those rounds. Expanding the target age range of the two SIAs in 2012 without the improved coverage for the under-vaccinated subpopulation leads to less reduction in paralytic cases and elimination time (Figure 3a and Table 4).

Looking prospectively, with very few cases predicted by the model for the second half of 2013 and 2014, Figure 3b focuses on incidence since 2013. Figure 3b shows relatively little impact on WPV1 cases of an expanded age group SIA in late 2013, regardless of whether the round targets 0–14 year olds or all ages. Elimination in the model occurs 17 days earlier if the expanded age group SIA round targets 0–14 year olds and 49 days earlier if the expanded age group SIA targets all ages, at the costs of 9.3 or 29.6 million additional doses, respectively (Table 4). Even a moderate, but sustained, improvement in the relative SIA coverage for the under-vaccinated subpopulation by 0.05 (from 0.2) leads to a slightly greater reduction of elimination time than a single expanded age group SIA round, with 58 days earlier WPV1 elimination achieved in the model. This suggests that the impact of the expanded age group SIA currently remains limited given the existence of a preferentially mixing and under-vaccinated subpopulation, particularly given our assumption that the expanded age group SIA achieves coverage at the same very low rate we assume for regular SIAs that target 0–4 olds (i.e., a true coverage per round of only 0.2 × 0.85 = 0.17). We find similar results for variations of these scenarios with different timing of the expanded age group SIAs, two consecutive rounds, or annually repeated rounds, in part also because the assumed *status quo* trajectory does not lead to many expected future cases (i.e., assuming 11 rounds per year with sustained high quality in the general population).

## Discussion

The results from this analysis suggest that expanded age group SIAs may prevent polio cases and shift WPV elimination forward in time, in some cases significantly and in other cases moderately, depending on the circumstances. If conducted preventively before a possible surge in cases (e.g., prior to an introduction into a polio-free area, a time of poor vaccination intensity in an endemic area), then we expect the expanded age group round to prevent a significant number of cases. This supports the GPEI policy since 2012 of expanding the age group targeted in the first two SIAs in response to an outbreak in a polio-free country independent of the initial age distribution of cases, as long as the outbreak response remains large enough to include areas not yet affected by the outbreak. The ability of reactive expanded age group SIAs to stop transmission in endemic areas depends on the target age range and how well the expanded age group round reaches the key reservoirs of transmission. Consequently, the existence of a preferentially mixing subpopulation consisting of clusters of un(der)-vaccinated individuals in large enough numbers to sustain transmission represents a key limiting factor for expanded age group rounds if these rounds do not reach these populations better than regular SIAs targeting children under 5 years of age. However, the model results show that as new under-vaccinated communities get identified with improved micro-planning, targeting age groups older than only children under 5 years of age in these communities may under some circumstances help accelerate the interruption of WPV transmission. Moreover, the widespread social mobilization of a massive expanded age group round may help to reach more children under 5 years of age as well. Vaccinating expanded age groups immunizes some of the remaining fully susceptible individuals in these age groups, which although they represent only a small proportion of all individuals in these age groups [33], could add up to large numbers due to the size of the population of individuals over 4 years of age.

Perhaps more importantly, vaccinating the expanded age groups boosts already immune individuals so that their ability to participate in transmission decreases. The importance of this effect depends on the inherent effect of waning on the ability to participate in poliovirus transmission. While some consensus suggests that even individuals with historic poliovirus infection or vaccination and serum antibodies that dropped below the detection limit still remain protected from paralytic poliomyelitis disease due to memory immunity, much uncertainty remains about the role of memory immunity in limiting participation in asymptomatic poliovirus transmission [21,22]. Several studies suggest that individuals with historic immunity become infected as readily after an OPV challenge as fully susceptible individuals [34-38], but data on duration and titers of excretion remain limited and no single study provides a direct comparison between known fully susceptible individuals and individuals with historic, waned immunity [21]. Moreover, the relationship between any reduction in excreted virus titers and infectiousness to others remains highly uncertain due to the practical challenges of measuring these directly [22]. Nevertheless, the epidemiology of polio strongly suggests that memory immunity reduces participation in transmission to some extent, because otherwise childhood vaccination would not sustain wild polio-free status in so many countries in the world for such a long time. Our assumptions about waning represent the result of an extensive model calibration process within uncertainty bounds obtained from expert assessments [22] based on careful consideration of the literature [21] that produced dynamic poliovirus transmission behavior consistent with the evidence across a large number of situations, including Tajikistan, northern India, and northwestern Nigeria [20]. Specifically, we assume that individuals with 2 or more historic poliovirus infections (from any live poliovirus infection) ultimately acquire a relative fecal-oral transmission potential compared to fully susceptible individuals of approximately 0.1 after starting out with a relative fecal-oral transmission potential of near 0 immediately after recovery from the infection. The assumptions differ by immunity state (e.g., for individuals with only 1 prior poliovirus infection, we assume approximately double these values; see Additional file 1). The expanded age group rounds move individuals from the historic to the recent immunity state, which explains the improvement in the overall population immunity even if very few fully susceptible individuals remain in the older age groups. We examined different assumptions about waning but those would produce behavior inconsistent with the evidence in some situations, unless we modified other assumptions. For example, in northwestern Nigeria, with more waning arguably under-vaccinated subpopulations matter less and the general population may sustain WPV transmission on its own, such that expanded age group SIAs could yield more benefits (i.e., reduced cases and shorter duration of transmission). However, such a scenario produces many more cases during the early 2000s, which appears inconsistent with the evidence, and would also imply that widespread WPV transmission continues throughout northwestern Nigeria even after 2010, rather than transmission confined to clusters of preferentially mixing under-vaccinated communities. Based on the extensive model calibration process, we believe that our model provides a realistic characterization of waning. While many existing poliovirus transmission models ignore the potentially important dynamics of reinfection and waning, one model that includes it also assumed increasing potential to participate in transmission as a function of time since the last infection [39]. Compared to our assumption of 0.1, Mayer et al. assumed that waning immunity eventually leads to a much higher relative transmission potential compared to fully susceptible individuals of between approximately 0.2 and 0.6, based on their interpretation of the evidence without a direct comparison to poliovirus incidence data [39].

Our prospective results for northwestern Nigeria offer some hope that the area we modeled may achieve sufficient population immunity to stop WPV1 elimination by the end of 2014 given the assumed current path. However, this part of Nigeria represents only one part of the country, which must achieve and maintain high levels of vaccine coverage and population immunity everywhere. The results shown here depend on the continuation of the aggressive immunization strategy that we modeled, and any change in strategy will change the results. Continued insecurity in the region, particularly in Kano state, represents an ongoing concern, which might reduce the ability to sustain immunization intensity in the area. Moreover, with only two tOPV SIAs per year the model did not project elimination of type 2 cVDPVs despite the assumed sustained improvement of SIAs going forward. This implies the need to actively manage population immunity for all three serotypes rather than focusing on a single serotype to reduce the immediate risks of importation outbreaks and cVDPV ermergence and the risks of type 2 cVDPV emergence following the planned cessation of type 2-containing OPV use [4,8,40,41]. Although we do not anticipate that our overall inferences related to the potential role of expanded age groups for SIAs would change significantly if a resurgence occurs, we emphasize that targeting expanded age groups may produce the largest reductions in expected cases and elimination times in areas with significant existing population immunity gaps. Although we focused on the northwestern part of Nigeria, the northeastern parts also warrant additional attention.

Our analyses yielded some important insights not directly related to SIAs targeting expanded age groups. First, the retrospective analysis of the 2010 Tajikistan outbreak makes a very strong case for a focus on prevention (i.e., more pSIAs), which could save both substantial health and financial costs. Given that an eradication initiative should focus on prevention of cases before they occur [4], pSIAs must represent an important programmatic priority. In the context of limited resources, continued assessment of population immunity in previously polio-free countries and identification of clusters of susceptible people should motivate efforts to conduct pSIAs [42]. Complementing serologic assessments and vaccination data collection, modeling may offer a tool to better identify population immunity gaps before they occur [4] and to determine the most important factors that contribute to low population immunity [43]. As long as WPV continues to circulate globally it can transmit across long distances [44], and the risk exists that the virus will find such clusters [45], countries will need to invest in both addressing these population immunity gaps and interrupting WPV globally as soon as possible. This suggests that if known immunity gaps exist, conducting preventive SIAs may offer a strategy that will save overall cases and doses of vaccine. Second, continued efforts to reach previously-missed or under-vaccinated populations remain essential to interrupting transmission, regardless of whether they focus only on children under 5 years of age or expanded age groups. However, reaching these groups with sufficient doses takes time and focusing only on these groups while ignoring the general population carries the risk of perpetually chasing the virus, without attaining enough general population immunity to prevent immunity gaps elsewhere. Thus, continued intense efforts to vaccinate the general populations everywhere remain critical while national programs in the remaining endemic countries continue to find and reach any remaining underserved populations.

As with any model, our results reflect the limitations of the model and the process used to generate model inputs. We rely on the approach of developing appropriate reference cases and making comparisons to these, to reduce the potential impact of the assumptions that we made to deal with inadequate data and multiple uncertainties as we calibrated the model. Our characterization of a subpopulation of un(der)-vaccinated individuals provides a tractable way to attempt to capture some of the complex heterogeneity that exists, but we recognize that it represents an abstract construction and that real populations are highly complex systems. Although we attempt to capture all of the important complexities that exist, our model represents a simplification of reality. With respect to the interpretation of the model results for the time to elimination, we caution that our simplistic approach to incorporate die-out in our deterministic model does not capture the stochastic processes and true heterogeneity that affect real elimination of polioviruses. Thus, the shifts in elimination times between scenarios that we reported indicate differences in the time when WPV prevalence in the last remaining mixing group reach very low levels, which may not fully capture all of the uncertainty and variability that underlies the actual process of die out. Other limitations related to the differential-equation based model discussed in detail elsewhere also apply to this analysis of expanded age groups [20].

## Conclusions

This study suggests the need to carefully understand the epidemiological situation, including the role of subpopulations of susceptible individuals, and the potential for waning immunity and re-infection of older children and adults. Our results suggest relatively modest benefits of expanded age group SIAs on estimated paralytic cases and the time of interruption of WPV transmission, with the greatest potential to increase population immunity and accelerate WPV eradication for SIAs conducted preventively or reaching under-vaccinated sub-groups.

## Abbreviations

AFP: Acute flaccid paralysis; bOPV: Type 1 and 3 bivalent OPV; cVDPV: Circulating vaccine-derived poliovirus; DTP: Difteria-tetanus-pertussis vaccine; GPEI: Global polio eradication initiative; IPV: Inactivated poliovirus vaccine; LPV: Live poliovirus; mOPV1,3: Type 1 and 3 monovalent OPV, respectively; NA: Not applicable; NPAFP: Non-polio AFP; OPV: Oral poliovirus vaccine (generic term); oSIA: Outbreak response SIA; POL: Any polio vaccine; pSIA: Preventive SIA; PV1,2,3: Type 1, 2, or 3 poliovirus (generic terms); R0: Basic reproductive number; SIA: Supplemental immunization activity; tOPV: Trivalent OPV; WPV: Wild poliovirus (generic term); WPV1,2,3: WPV type 1, 2, or 3, respectively; WUP: Western Uttar Pradesh.

## Competing interests

The authors declare that they have no competing interests.

## Authors’ contributions

RJDT led the development and implementation of the poliovirus transmission model and expanded age group scenarios, conducted the analyses for Tajikistan and northwestern Nigeria, and drafted the manuscript. DAK participated in the development of scenarios and implemented and conducted the analysis for northern India. SGFW, MAP, and SLC participated in the development of expanded age groups scenarios and helped with interpretation of the data for each situation. KMT conceived of the study, participated in its design and coordination and helped draft the manuscript. All authors revised the manuscript and read and approved the final manuscript.

## Pre-publication history

The pre-publication history for this paper can be accessed here:

http://www.biomedcentral.com/1471-2334/14/45/prepub

## Supplementary Material

Additional file 1 Technical appendix. Click here for file

## References

[B1] Sutter RW Kew OM Cochi SL Plotkin SA, Orenstein WA, Offit PA Poliovirus vaccine -- live Vaccines. Fifth edition 2008 Philadelphia: Saunders Elsevier

[B2] World Health Organization Transmission of wild poliovirus type 2 - apparent global interruption Wkly Epidemiol Rec 2001 14 95 97 11315462

[B3] Global Polio Eradication Initiative - list of wild poliovirus by country http://www.polioeradication.org/Dataandmonitoring/Poliothisweek/Wildpolioviruslist.aspx

[B4] Thompson KM Pallansch MA Duintjer Tebbens RJ Wassilak SGF Cochi SL Modeling population immunity to support efforts to end the transmission of live polioviruses Risk Anal 2013 14 647 663 10.1111/j.1539-6924.2012.01891.x 22985171 PMC7896539

[B5] World Health Assembly Poliomyelitis: intensification of the global eradication initiative (resolution 65.5) 2012 Geneva, Switzerland: World Health Organization

[B6] Thompson KM Pallansch MA Duintjer Tebbens RJ Wassilak SG Kim J-H Cochi SL Pre-eradication vaccine policy options for poliovirus infection and disease control Risk Anal 2013 14 516 543 10.1111/risa.12019 23461599 PMC7941951

[B7] Hull HF Ward NA Hull BP Milstien J de Quadros CA Paralytic poliomyelitis: seasoned strategies, disappearing disease Lancet 1994 14 1331 1337 10.1016/S0140-6736(94)92472-4 7910329

[B8] World Health Organization Global polio eradication initiative: polio eradication and endgame strategic plan (2013–2018) 2013 Geneva, Switzerland: World Health Organization Report No.: WHO/POLIO/13.02

[B9] Sabin AB Ramos-Alvarez M Alvarez-Amezquita J Pelon W Michaels RH Spigland I Koch MA Barnes JM JSR Live, orally given poliovirus vaccine: effects of rapid mass immunization on population under conditions of massive enteric infection with other viruses JAMA 1960 14 1521 1526 10.1001/jama.1960.03020320001001 14440553

[B10] Olive JM Risi JB Jr de Quadros CA National immunization days: experience in Latin America J Infect Dis 1997 14 1 S189 S193 9203715 10.1093/infdis/175.supplement_1.s189

[B11] Más Lago P Eradication of poliomyelitis in Cuba: a historical perspective Bull World Health Organ 1999 14 681 687 10516790 PMC2557718

[B12] Risi JB Jr The control of poliomyelitis in Brazil Rev Infect Dis 1984 14 S400 S403 10.1093/clinids/6.Supplement_2.S400 6740081

[B13] Prevots DR Atti ML C d Sallabanda A Diamanti E Aylward RB Kakariqqi E Fiore L Ylli A van der Avoort HG Sutter RW Outbreak of paralytic poliomyelitis in Albania, 1996: high attack rate among adults and apparent interruption of transmission following nationwide mass vaccination Clin Infect Dis 1998 14 419 425 10.1086/516312 9502465

[B14] Centers for Disease Control and Prevention Outbreaks following wild poliovirus importations --- Europe, Africa, and Asia, January 2009-September 2010 Morb Mortal Wkly Rep 2010 14 1393 1399 21048560

[B15] Gregory CJ Ndiaye S Patel M Hakizamana E Wannemuehler K Ndinga E Chu S Talani P Kretsinger K Investigation of elevated case-fatality rate in poliomyelitis outbreak in Pointe Noire, Republic of Congo, 2010 Clin Infect Dis 2012 14 1299 1306 10.1093/cid/cis715 22911644

[B16] World Health Organization Outbreak news. Poliomyelitis, Namibia Wkly Epidemiol Rec 2006 14 238 16783892

[B17] Oostvogel P van Wijngaarden J van der Avoort HG Mulders MN Conyn-van Spaendonck MA Rümke H van Steenis G van Loon AM Poliomyelitis outbreak in an unvaccinated community in The Netherlands, 1992–3 Lancet 1994 14 665 670 10.1016/S0140-6736(94)92091-5 7915354

[B18] Thompson KM Duintjer Tebbens RJ Eradication versus control for poliomyelitis: an economic analysis Lancet 2007 14 1363 1371 10.1016/S0140-6736(07)60532-7 17448822

[B19] Duintjer Tebbens RJ Pallansch MA Cochi SL Wassilak SGF Linkins J Sutter RW Aylward RB Thompson KM Economic analysis of the global polio eradication initiative Vaccine 2011 14 334 343 10.1016/j.vaccine.2010.10.026 21029809

[B20] Duintjer Tebbens RJ Pallansch MA Kalkowska DA Wassilak SG Cochi SL Thompson KM Characterizing poliovirus transmission and evolution: insights from modeling experiences with wild and vaccine-related polioviruses Risk Anal 2013 14 703 749 23521018 10.1111/risa.12044 PMC11700012

[B21] Duintjer Tebbens RJ Pallansch MA Chumakov KM Halsey NA Hovi T Minor PD Modlin JF Patriarca PA Sutter RW Wright PF Expert review on poliovirus immunity and transmission Risk Anal 2013 14 544 605 10.1111/j.1539-6924.2012.01864.x 22804479 PMC7896540

[B22] Duintjer Tebbens RJ Pallansch MA Chumakov KM Halsey NA Hovi T Minor PD Modlin JF Patriarca PA Sutter RW Wright PF Review and assessment of poliovirus immunity and transmission: synthesis of knowledge gaps and identification of research needs Risk Anal 2013 14 606 646 10.1111/risa.12031 23550968 PMC7890644

[B23] Sterman J Business dynamics: systems thinking and modeling for a complex world 2001 Boston, MA: McGraw-Hill

[B24] Lloyd AL Realistic distributions of infectious periods in epidemic models: changing patterns of persistence and dynamics Theor Popul Biol 2001 14 59 71 10.1006/tpbi.2001.1525 11589638

[B25] Duintjer Tebbens RJ Pallansch MA Kim J-H Burns CC Kew OM Oberste MS Diop O Wassilak SGF Cochi SL Thompson KM Review: oral poliovirus vaccine evolution and insights relevant to modeling the risks of circulating vaccine-derived polioviruses (cVDPVs) Risk Anal 2013 14 680 702 23470192 10.1111/risa.12022 PMC7890645

[B26] **World population prospects: the 2010 revision** http://esa.un.org/unpd/wpp/Documentation/WPP%202010%20publications.htm

[B27] World population prospects: the 2012 revision http://esa.un.org/unpd/wpp/index.htm

[B28] Centers for Disease Control and Prevention Update on vaccine-derived polioviruses--worldwide, July 2009-March 2011 MMWR Morb Mortal Wkly Rep 2011 14 846 850 21716199

[B29] World Health Organization Summary of discussions and recommendations, 16th informal consultation of the global polio laboratory network, 22–23 September 2010, WHO/HQ 2010 Geneva, Switzerland: World Health Organization

[B30] Wassilak SGF Pate MA Wannemuehler K Jenks J Burns C Chenoweth P Abanida EA Adu F Baba M Gasasira A Outbreak of type 2 vaccine-derived poliovirus in Nigeria: emergence and widespread circulation in an underimmunized population J Infect Dis 2011 14 898 909 10.1093/infdis/jiq140 21402542 PMC3068031

[B31] Nathanson N Kew OM From emergence to eradication: the epidemiology of poliomyelitis deconstructed Am J Epidemiol 2010 14 1213 1229 10.1093/aje/kwq320 20978089 PMC2991634

[B32] Thompson KM Duintjer Tebbens RJ Pallansch MA Evaluation of response scenarios to potential polio outbreaks using mathematical models Risk Anal 2006 14 1541 1556 10.1111/j.1539-6924.2006.00843.x 17184396

[B33] Giwa FJ Olayinka AT Ogunshola FT Seroprevalence of poliovirus antibodies amongst children in Zaria, Northern Nigeria Vaccine 2012 14 6759 6765 10.1016/j.vaccine.2012.09.023 23000220

[B34] Abbink F Buisman AM Doornbos G Woldman J Kimman TG Conyn-van Spaendonck MAE Poliovirus-specific memory immunity in seronegative elderly people does not protect against virus excretion J Infect Dis 2005 14 990 999 10.1086/427810 15717277

[B35] Glezen PW McCollough RH Lamb GA Chin TDY Quantitative relationship of preexisting homotypic antibodies to excretion of poliovirus types 1, 2, and 3 following the feeding of trivalent attenuated poliovirus vaccine Am J Epidemiol 1969 14 146 156 4308600 10.1093/oxfordjournals.aje.a121058

[B36] Glezen WP Lamb GA Belden EA Chin TD Quantitative relationship of preexisting homotypic antibodies to the excretion of attenuated poliovirus type 1 Am J Epidemiol 1966 14 224 237 5930775 10.1093/oxfordjournals.aje.a120578

[B37] Smith JWG Lee JA Fletcher WB Yetts R Magrath DI Perkins FT The response to oral poliovaccine in persons aged 16–18 years Journal of Hygiene Cambridge 1976 14 235 247 10.1017/S0022172400055133 PMC2129633 177700

[B38] Siegert R Enders-Ruckle G Oehme J Waller D Der einfluß der prävakzinalen immunität auf die virusausscheidung und antikörperproduktion nach polio-schluckimpfung mit Typ I (Sabin) [the effect of prevaccinal immunity on virus excretion and antibody production after polio immunization with type I (Sabin)] Deutsche medizinische Wochenschrift 1963 14 1586 1594 10.1055/s-0028-1112269 14047334

[B39] Mayer BT Eisenberg JN Henry CJ Gomes MG Ionides EL Koopman JS Successes and shortcomings of polio eradication: a transmission modeling analysis Am J Epidemiol 2013 14 1236 1245 10.1093/aje/kws378 23592542 PMC3664334

[B40] Kalkowska DA Duintjer Tebbens RJ Thompson KM Modeling strategies to increase population immunity and prevent poliovirus transmission in the high-risk area of northwest Nigeria J Infect Dis 2014 (In press). doi:10.1093/infdis/jit834 10.1093/infdis/jit834 25316863

[B41] Thompson KM Duintjer Tebbens RJ Current polio global eradication and control policy options: perspectives from modeling and prerequisites for OPV cessation Expert Reviews of Vaccines 2012 14 449 459 10.1586/erv.11.195 22551030

[B42] Lowther SA Roesel S O’Connor P Landaverde M Oblapenko G Deshevoi S Ajay G Buff A Safwat H Salla M World Health Organization regional assessments of the risks of poliovirus outbreaks Risk Anal 2013 14 664 679 10.1111/risa.12032 23520991

[B43] Thompson KM Duintjer Tebbens RJ Modeling the dynamics of oral poliovirus vaccine cessation J Infect Dis 2014 (In press). doi:10.1093/infdis/jit845 10.1093/infdis/jit845 25316870

[B44] Kidd S Goodson JL Aramburu J Morais A Gaye A Wannemuehler K Buffington J Gerber S Wassilak S Uzicanin A Poliomyelitis outbreaks in Angola genetically linked to India: risk factors and implications for prevention of outbreaks due to wild poliovirus importations Vaccine 2011 14 3760 3766 10.1016/j.vaccine.2011.03.034 21440639

[B45] Centers for Disease C, Prevention Notes from the field: outbreak of poliomyelitis--Somalia and Kenya, May 2013 Morb Mortal Wkly Rep 2013 14 484 PMC4604849 23760191

