# Supplementary material for: The potential impact of expanding target age groups for polio immunization campaigns
Source: BMC Infect Dis. 2014 Jan 29;14:45. doi: 10.1186/1471-2334-14-45 (PMC3918103; doi:10.1186/1471-2334-14-45)
Supplement: Additional file 1 — Technical appendix. [file 1471-2334-14-45-S1.doc]

**TECHNICAL APPENDIX**

**A1. Equations for age-mixing and sub-population mixing**

Given the importance of age-mixing and mixing between subpopulations for the analysis of expanded age group SIAs, this section summarizes our equations, as described in more detail in prior work.[[1](#_ENREF_1), [2](#_ENREF_2)] For simplicity, we use the shorthand notation of the effective proportion infectious (EPI) for each mixing age group in the model, which represents the prevalence of infection weighted by the relative contribution to transmission of individuals by immunity state.[[1](#_ENREF_1)] In the model, this quantity exists for each virus strain and depends on the mode of transmission (fecal-oral or oropharyngeal) and the relative infectiousness in each infection stage for individuals depending on their prior immunity state. It may represent a sum over multiple model age groups since we always use the same mixing age groups of 0-4 years, 5-14 years, and 15 or more years while the model age groups typically remain narrower.

For age-mixing, we use the preferential mixing model, with *κ*(*a*) representing the proportion of contacts in mixing age groups a reserved for other individuals in mixing age group *a,* while the remaining proportion 1- *κ*(*a*) of contacts get evenly distributed over all other age groups, including age group *a*.[[3](#_ENREF_3), [4](#_ENREF_4)] For the three situations considered in this analysis, we assume constant κ by mixing age group, with *κ* of 30%, 35%, and 40% of contacts in Tajikistan, northern India, and northwestern Nigeria, respectively, as noted in the text. Based on standard theory for preferential mixing,[[3](#_ENREF_3)] the normalized mixing matrix *M*(*a*,*b*) equals:

$$M(a,b)(t)={\kappa(a)1}_{\{a=b\}}+\frac{\left( 1-\kappa\left( a \right) \right)\left( 1-\kappa\left( b \right) \right)N_{b}(t)}{\sum_{c=0}^{n} N_{c}(t)\left( 1-\kappa\left( c \right) \right)}$$

where *N_a_*(*t*) represents the number of people in mixing age group *a* at time *t* and the indicator function 1_{condition}_ equals 1 if the condition holds or 0 otherwise. Given that N depends on time, the mixing matrix gets recalculated at each time step.

For mixing between sub-populations, we use a similar but slightly different construct. We hypothetically divide the model population into *m* subpopulations of equal size. We consider one of these *m* subpopulations the under-vaccinated subpopulation, while the remaining *m*-1 subpopulations represent the general populations. We then define *p*_within_ as the proportion of contacts of any of the *m* subpopulations reserved for individuals of the same subpopulation, with the remaining proportion 1- *p*_within_ occurring with the other *m*-1 subpopulations, not including the given subpopulation. Thus, for individuals in the under-vaccinated subpopulation, the weight for contacts from the same under-vaccinated subpopulation equals *p*_within_ and the weight for contacts from the general population equals 1- *p*_within_.

Combining the expressions for age-mixing and sub-population mixing, we obtain the following expression for the force-of-infection from a given virus strain to an individual in mixing age group *a* of the under-vaccinated subpopulation:

$$\lambda_{a}^{sub}\left( t \right)=\beta\sum_{b=1}^{{na}_{m}-1} {EPI}_{b}^{sub}\left( t \right)M^{sub}(a,b)(t)p_{within}+{EPI}_{b}^{gen}\left( t \right)M^{gen}(a,b){(1-p}_{within})$$

Here, *na_m_* refers to the number of mixing age groups (i.e., 3 for all analyses in this paper), *β* represents the approximate transmission coefficient which in the model depends on *R_0_*, the mortality rate and the transmission mode-dependent average duration of the infectious period for fully susceptible individuals,[[1](#_ENREF_1)] and the subscripts ‘sub’ and ‘gen’ denoted the under-vaccinated subpopulation and the general population, respectively. In our model, the mixing matrices *M^sub^*(*a,b*) and *M^gen^*(*a,b*) remain the same because we do not assume any differences in demographic inputs for the two subpopulations.

For the general population, all of the *p*_within_ within-subpopulation contacts occur with members within the general population. However, we must divide the proportion 1- *p*_within_ of outside-subpopulation contacts to include (*m*-2)/(*m*-1) contacts with members of other subpopulations within the general population and 1/(*m*-1) contacts with members of the under-vaccinated subpopulation. Thus, for individuals in the general subpopulation, the weight for contacts from the general population equals *p*_within_+ (1-*p_within_*)× (*m*-2)/(*m*-1) and the weight for contacts from the under-vaccinated subpopulation equals (1- *p*_within_)/(*m*-1). For individuals in the general population, the expression for the force-of-infection equals:

$\lambda_{a}^{gen}\left( t \right)=\beta\sum_{b=1}^{{na}_{m}-1} {EPI}_{b}^{sub}\left( t \right)M^{sub}(a,b)(t)(1-p_{within})/(m-1)+{EPI}_{b}^{gen}\left( t \right)M^{gen}\left( a,b \right)\left( {(1-p}_{within})(m-2)/(m-1)+p_{within} \right)$

To characterize die-out in the model, λ for any subpopulation and age group becomes 0 if the weighted sum of the effective proportion infectious drops below the transmission threshold (i.e., of 5 per million).

Figure A1 shows the relative contribution to transmission of individuals with recent or historic LPV infections, defined as the product of their relative susceptibility, relative infectiousness, and relative duration of infectiousness compared to fully susceptible individuals.[[1](#_ENREF_1)]

**A2. Characterization of SIAs in the prospective model**

*Rationale*

Our approach to characterize historic SIAs for the retrospective model[[1](#_ENREF_1)] relied on specifying vaccination rates that produced realistic proportions of missed children by SIAs after each calendar year, based on the assumption that every targeted individual in any subpopulation faced an equal chance of receiving a dose during each round.[[1](#_ENREF_1)] Consequently, as the number of rounds in a year increased, the approach required decreasing the effective per-round impacts (denoted with ζ in the article[[1](#_ENREF_1)]) to achieve the same annual cumulative percentage of missed children by SIAs. For example, to accomplish 10% cumulatively missed children by SIAs in a given year with 3 rounds required an effective per-round impact of ζ = 54% while with 6 rounds this decreased to ζ = 32%. However, in the extreme event that 90% of targeted children simply received 6 doses while 10% received none, this would imply a true coverage of 90% in each round. This extreme event would imply many more doses used, the majority reaching recently vaccinated and immune children. In most real situations, the reality probably lies between these two examples. Our simplified approach for the retrospective model[[1](#_ENREF_1)] implicitly assumed that the doses received by already vaccinated children do not affect the dynamics significantly. However, we recognize that the simplification we used to fit the generic model inputs will not support prospective analyses to directly test different assumptions about the impact of individual rounds as opposed to the cumulative impact over some specified time period (e.g., a calendar year). Thus, we developed a characterization of SIAs for the prospective model that allows direct specification of the true coverage of individual rounds and the probability of children repeatedly receiving or missing doses. The characterization allows direct extraction of the number of doses administered, which translates into doses distributed after accounting for wastage, and comparison of zero-dose proportions in the model to the reported zero-dose proportions among NPAFP cases.

*General characterization*

Ideally, characterization of SIAs would specify probabilities of a targeted child receiving a dose during a round conditional on any possible vaccination history (from routine and SIAs). This would capture the reality that hard to reach or underserved individuals based on past rounds may experience a higher risk of not receiving a dose than individuals who received doses in most or all of their dose opportunities. However, our differential-equation based model tracks immunity states for individuals in aggregate and not individual dose histories, which remain different because individuals can become immune without receiving a dose (i.e., through WPV or secondary OPV infection) or receive a dose without becoming immune due to imperfect take of all polio vaccines.[[1](#_ENREF_1), [5](#_ENREF_5)] Stratifying the model by all possible dose histories would add more complexity to the model than practically workable (since in some situations children receive more than 20 doses of different OPVs, for example in northern India). Even if we could track dose histories in more detail (e.g., using an individual-based model), insufficient data exist to support specification of the conditional probabilities of receiving a dose during an SIA by all possible dose histories. Therefore, our approach focuses on conditional probabilities of receiving a SIA dose depending only on receipt of a dose in the previous round. Specifically, the approach specifies the following new model inputs, all bounded between 0 and 1:

- The true coverage (*TC*) of an SIA round, defined as the fraction of the targeted population that receives a dose in a given round.
- The repeated missed probability (*P_RM_*), defined as the conditional probability that a targeted individual does not receive a dose in a round, given that the individual did not receive a dose in the previous round despite falling into the targeted population for that round.
- The repeated reached probability (*P_RR_*), defined as the conditional probability that a targeted individual receives a dose in a round, given that the individual received a dose in the previous round.

*TC* depends on the size of the target population (*N*), the number of doses distributed (*ND*), and the wastage factor (*w*), defined as the fraction of doses distributed to the field that does not get administered:

*TC* = *ND*×(1-*w*)/*N*

*TC* typically gets measured by administrative data on doses distributed and by campaign monitoring, or sometimes by surveys conducted after individual SIAs (e.g., lot quality assessment surveys). *P_RM_* and *P_RR_* capture the likely reality of a correlation of receiving doses or not in subsequent rounds. Given that *P_RM_* and *P_RR_* must together produce *TC*, specification of two of the three model inputs above suffices to characterize an SIA (as illustrated in Figure A2), which considers two consecutive rounds. In Figure A2, branch b1 represents the fraction of targeted individuals who receive a dose in two consecutive rounds, b2 those who receive a dose in the first but not in the second round, b3 those who receive a dose in the second but not in the first round, and b4 those who do not receive a dose in either round. The total fraction who receives a dose in the second round equals:

*TC_2_* = b1 + b3 = *TC_1_*×*P_RR_* + (1-*TC_1_*)×(1-*P_RM_*)

Where *TC_i_* denotes the true coverage of round *i* and *P_RR_* and *P*_RM_ both pertain to the second round. Thus, *P_RR_* must satisfy:

*P_RR_* = (*TC_2_* - (1-*TC_1_*)×(1-*P_RM_*))/*TC_1_*

We note that if *TC* remains equal between successive rounds, then *P_RR­_* remains in the interval [0,1] for any values of *TC* and *P_RM_*, but the more *TC* changes between successive rounds, the more limits exist on *P_RM_* to keep *P­_RR_* in the interval [0,1]. For example, *TC_1_*=0.75 and *TC_2_*=0.80 leads to the requirement of *P_RM_*<0.73.

*Use in the model*

To apply the above characterization in the model, we must keep track of the fraction of the population in each immunity state that received a dose in the most recent round. For simplicity, we do so only for those individuals who did not yet acquire active immunity (i.e., from vaccination or natural exposure to a LPV), which represent the main drivers of immunity. Specifically, we divide all of the fully susceptible and maternally immune individuals (FSMI) into three categories, each subject to the appropriate probabilities of receiving a dose if exposed to an SIA and still falling within the target age range:

- New children (*NC,* as fraction of all targeted children) born after the previous SIA round who receive a dose in the current round with probability *TC*.
- Reached children (*RC,* as fraction of all targeted children) who received a dose in the previous SIA round but remained FSMI due to failure to take and who receive a dose in the current round with probability *P_RR_*.
- Missed children (*MC,* as fraction of all targeted children) who did not receive a dose in the previous SIA round and who receive a dose in the current round with probability 1-*P_RM_*.

To determine the vaccination rates for all targeted FSMIs, we use the average coverage for all FSMIs:

*cov_FSMI_* = *TC*×*NC +P_RR_*×*RC +* (1-*P_RM_*)×*MC*

To determine the fraction of FSMIs in each of the above three categories (by age), we begin to accumulate new FSMIs from newborns as soon as any SIA round finishes in age-dependent stocks for new fully susceptible individuals (i.e., *NFS_a_*(*t*)) and new maternally immune individuals (i.e., *NMI_a_*(*t*)), subject to the same in- and outflows as any other fully susceptible individuals and maternally immunes in the model. Thus, for age group *a* and at the beginning of the current SIA (i.e., time *t_curr_*):

*NC_a_* = (*NFS_a_*(*t_curr_*)+ *NMI_a_*(*t_curr_*))/(*FS_a_*(*t_curr_*)*+MI_a_*(*t_curr_*)).

where *FS_a_* and *MI_a_* represent the total number of fully susceptible and maternally immune individuals in age group *a*, respectively. The remaining fraction of FSMIs represents either *MC* or *RC*. To determine the breakdown of all remaining *FSMI*s into *MC* and *RC*, the model “remembers” those fractions from the previous round. Given that both *MC* and *RC* behave as fully susceptible or maternally immune individuals, they remain subject to the same fractional outflows between subsequent rounds, and therefore the fractions remain intact. Specifically, for given age group *a,* the fractions equal:

*MC*/(*MC*+*RC*) = (1-*cov_FSMI_*)/(1- *tr*×*cov_FSMI_*)^σi^ (1)

*RC*/(*MC*+*RC*)= 1- *MC*/(*MC*+*RC*) (2)

Here, *tr* represents the appropriate take rate for the vaccine used during the previous round, and σ_i_ the relative susceptibility of the respective immunity state, which equals 1 for fully susceptible and approximately 0.8 for maternally immune individuals.[[1](#_ENREF_1)] The fraction of 0.8 accounts for the assumed lower susceptibility to live poliovirus for maternally immune than fully susceptible individuals. In the model, this translates into multiplication of the effective vaccination rate due to the SIA by relative susceptibility, leaving more maternally immune than fully susceptible recipients of a dose uninfected. Consequently, the fractions of remaining children missed (i.e., *MC*/(*MC*+*RC*) or that did not take (i.e., *RC*/(*MC*+*RC*)) differ slightly between fully susceptible and maternally immune individuals.

Finally, we compute the coverage *cov_Imm_* for all individuals with actively acquired immunity (i.e., those not fully susceptible or maternally immune at the beginning of an SIA round) based on the requirement that the overall coverage equals *TC_._*

*cov_Imm_* = (*TC* – *fsmi*×*cov_FSMI_)/*(1- *fsmi*)

where *fsmi* denotes the fully susceptible or maternally immune proportion of the target population. We multiply both *cov_FSMI_* and *cov_Imm_* by the fraction *F* of all individuals in the modeled population within target age range that an SIA targets. *F* typically equals 1 but may equal less than 1 if the modeled population represents an entire state while the SIA targets only a subset of all districts in the state (i.e., fractional rounds).

We calculate effective vaccination rates as *evr_FSMI_* =-ln(1-cov_FSMI_×*tr*)/*d* for FSMIs and *evr_FSMI_* =-ln(1-cov_Imm_×*tr*)/*d* for immunes, where *tr* is the appropriate take rate for the SIA and *d* the duration of the SIA, similar to the retrospective model[[1](#_ENREF_1)] except for the dependence on the immunity state. As in the retrospective model,[[1](#_ENREF_1)] these effective vaccination rates change over time according to the dates and assumed *TC* and *P_RM_* for each SIA, but remain constant for the duration of each round.

*Calculation of implied zero-dose children*

To interpret model assumptions about the true coverage and repeated miss probabilities of SIAs in a given situation, we derive the zero-dose proportions implied by those inputs. Doing so allows comparison to existing data about dose histories of children in population, in particular to the zero-dose proportions reported among NPAFP cases recorded as part of the AFP surveillance reporting system. Thus, we need to estimate the probability that a child receives neither routine nor SIA doses, given assumed true coverage, repeated miss probabilities, and routine immunization coverage levels. Given that meaningful data on zero-dose proportions only exist for young children, we consider only children that did not yet reach the upper end of the target age range (i.e., typically 5 years of age).

We start with the probability of not receiving any SIA dose by defining the following Bernoulli event for given child *x*:

A = “Child *x* did not receive any SIA doses at time *t*” with probability P(A)

While the formula for P(A) is straightforward when all SIAs target the entire modeled population, a complication arises when SIAs target only a fraction of all individuals in the target age range from the modeled population, which we refer to as fractional rounds. In the retrospective model, we characterized fractional rounds simply by multiplying the per-round impact by the targeted fraction of the modeled population.[[1](#_ENREF_1)] However, the concept of repeatedly missing individuals in rounds changes in the context of fractional rounds, because some individuals missed by the fractional round get missed not because they represent members of hard to reach or underserved communities, but because they simply fell outside of the targeted population. Therefore, we must consider three groups: targeted and reached, not targeted reachable, and truly missed. We refer to the last group as the *truly missed* (*TM*) fraction, while the second group represents the *omitted reachable* (*OR*) fraction (i.e., those missed in a round due to the fractional nature of the round). Individuals in both of these groups receive no vaccine in the current round, but we need to distinguish them because the probability of receiving a dose in a subsequent round depends on the status of truly missed (subject to repeated miss probability *P_RM_* ) or omitted reachable (subject to repeated reach probability *P_RR_*). Thus, we recursively calculate the zero-dose proportion after any given number of SIAs. After the first round, the expressions for the different groups equal:

*TM*_1­_ = (1 – *TC*_1_)

*OR*_1_ = (1 – *F*_1_) × *TC*_1_

For any subsequent round *r*, the recursive expressions equal:

*TM*_r­_ = *TM*_r-1_ × *P_RM_*_,r-1_

*OR*_r_ = *OR*_r-1_ × (1 – *F*_r_) × *P_RR_*_,r-1_+ *TM*_r-1_ × (1 – *F*_r_) × (1 – *P_RM_*_,r-1_)

Total zero-SIA-dose children after *nr* rounds = P(A) = *TM_nr_* + *OR_nr_*

Index *r* spans each SIA conducted in the modeled population of individual *x* ranging from first round after birth (*r*=1) to the most recent round that occurred at time *t* (*r*=*nr*). When all rounds target the entire modeled population (i.e., no fractional rounds such that all *OR_r_*=0), the expression reduces to:

$P(A)=(1-{TC}_{1})\times\prod_{r=2}^{nr} P_{RM,r}$

Consistent with this calculation of children missed by SIAs, in the model the missed children (*MC*, i.e., those subject to *P_RM_*) also includes truly missed children, while the reached children (*RC*, i.e., those subject to *P_RR_*) includes both reached and omitted reachable children (i.e., equations (1) and (2) in the previous subsection do not involve *F*).

With respect to routine immunization, coverage data typically characterize the coverage with the birth dose (*POL*_0_) separately from the coverage with each non-birth dose (*POL_i_*, *i =*1, 2, …). For simplicity, we assume that children who do not receive a first non-birth dose also do not receive a birth dose, so that we can directly estimate the probability for the event that a child did not receive any routine immunization dose from the routine immunization coverage data:

B = “Child *x* did not receive any routine doses” with probability P(B2) = 1-*POL_1_*(*t_birth+age1_*) (if the child is old enough to qualify for the first routine dose according to the recommended schedule, and 0 otherwise)

where *t_birth+age1_* indicates the time when child *x* reaches the age of the first dose according to the recommended schedule. The equation for P(B) assumes that all children who received at least one non-birth dose get counted towards *POL_1_*, even if they did not receive their first non-birth dose at the scheduled age of the first non-birth dose. This assumption remains consistent with the reported decreasing coverage estimates by dose[[6-8](#_ENREF_6)] (i.e., if children who received only a dose at the recommended age of the second dose would only get counted towards *POL*_2_, then *POL­_2_* coverage could exceed *POL_1_* coverage).

Finally, we similarly define the event:

C = “Child *x* did not receive any doses (whether routine or SIA)” with probability P(C)

P(C) is the probability that both A and B occur, or P(A ∩ B) (i.e., the probability of the conjunctive event “A ∩ B”). Following standard probability theory, P(A ∩ B) depends on the conditional probability that B occurs given that A occurs:

P(B) = P(A ∩ B) = P(A) × P(B | A)

P(B | A) specifies the conditional probability that child *x* did not receive a routine immunization dose given that (s)he did not receive an SIA dose. Similarly, one could compute P(A ∩ B) from P(A|B), but given our goal of characterizing missed children by SIAs, we focus on the former formula for P(B ∩ A). The conditional probability P(B | A) relates to the dependence between A and B. If A and B are independent, then P(B | A) = P(B). In contrast, if B completely and positively depends on A, then with probability 1 a child who did not receive any SIA doses also did not receive any routine immunization doses, so that P(B | A) = 1. Reasonably assuming positive dependence (i.e., the probability that a child did not receive any routine immunization dose given that the child did not receive any SIA dose equals or exceeds the unconditional probability that a child did not receive any routine immunization dose, P(B | A) ≥ P(B)), we can parameterize the range of values for P(B | A) as:

P(B | A) = P(B) + *dd* × (1-P(B))

where *dd* may range from 0 to 1 and it represents the degree of dependence between missing all SIA doses and missing all routine immunization doses, with *dd* = 0 corresponding to independence and *dd* = 1 to the greatest possible degree of positive dependence. We introduce this non-standard degree of dependence because the conventional (Pearson) correlation between two Bernoulli random variables cannot attain any arbitrary values in its range -1 to 1.[[9](#_ENREF_9)] For any given assumption about *dd*, substituting the above expression into equation 1 above gives the probability that child *x* did not receive any routine immunization or SIA dose:

P(C) = P(A ∩ B) = P(A) × P(B|A) = P(A) × (P(B) + *dd* × (1 - P(B))

P(C) represents the probability that child *x* received doses in the same sense as an NPAFP case reporting 0 doses. In the absence of any information on the dependence between routine immunization and SIA doses, we arbitrarily assume *dd*=0.5. We note that this choice does not impact the model results in any way, but only affects the comparison of implied zero-dose proportions in the model with available data and zero-dose children.

*SIA impact in under-vaccinated subpopulations*

In some situations (e.g., northern India, northwestern Nigeria), we characterize chronically under-vaccinated groups as separate subpopulations in the model.[[1](#_ENREF_1)] These may represent particularly hard-to-reach groups of people who get reached by SIAs at a much smaller overall coverage level than the general population. Moreover, we assume that these under-vaccinated groups preferentially mix with each other and consequently play an important role in sustaining WPV transmission even when coverage in the general population becomes very high. As AFP surveillance may also reach these groups at a much lower rate than the general population, we typically assume that zero-dose proportions from NPAFP cases reflect the SIA impact in the general population. We characterize the impact of SIAs based on a relative coverage level compared to the general population. Using superscripts “sub” and “gen” as above to denote the under-vaccinated subpopulation and the general population, respectively, we assume for any given SIA that:

*TC^sub^* = *TC^gen^*×*cov_rel_*

where *cov_rel_* denotes the relative coverage of the under-vaccinated subpopulation, which may change over time or by round. In general, multiplying the coverage for both previously reached and previously missed children by *cov_rel_* will not satisfy the requirement that these sum up to *TC*^sub^, and therefore we impose the somewhat arbitrary assumption that the coverage for previously reached and previously missed children both decrease by the same relative amount *r:*

*P_RR_^sub^* = *P_RR_^gen^*×*r*

1-*P_RM_^,sub^* = (1- *P_RM_^,gen^*)×*r*  → *P_RM_^e,sub^* = 1- (1- *P_RM_^gen^*)×*r*

For the under-vaccinated subpopulation the following equation must hold (i.e., the two top branches in Figure A2 still must add up to the true coverage):

*TC_2_^sub^* = *TC_1_^sub^*×*P_RR_^sub^* + (1-*TC_1_^sub^*)×*P_RM_^,sub^ = TC_1_^sub^*×*r*×*P_RR_^gen^* + (1-*TC_1_^sub^*) ×*r*×*P_RM_^,gen^*

Solving for *r*, we get:

*r* = *TC_2_^sub^*/ (*TC_1_^sub^*×*P_RR_^gen^* + (1-*TC_1_^sub^*) ×*P_RM_^,gen^*)

**A3. Model calibration**

Two factors significantly impact the calibration of the model: complexity and data quality. Our detailed description of the model[[1](#_ENREF_1)] provide context related to all of its various components, including the immunity states, the process of OPV evolution, and assumptions related to the dynamics of transmission (e.g., durations of infectiousness). We relied on extensive reviews of the literature and input from experts to develop the model, and we fix all inputs that should remain the same between different situations (i.e., inputs that depend on poliovirus serotypes or immunity states). We allow situation-specific inputs to vary appropriately. For example, we constrain values for R_0_ within reasonable ranges for each World Bank income level, and we use population data and estimates of routine immunization coverage from available sources. We face significant challenges with respect to data quality in the context of calibrating the model, because even with a global surveillance network, many cases do not get detected. We cannot observe infections that do not lead to cases, which means that we cannot observe the process of OPV evolution as it occurs. To calibrate the OPV evolution process in the absence of observable data, we compare the performance of the model to outbreaks with cVDPVs and to observations of no cVDPVs in situations in which they do not occur. By requiring the generic model inputs to work across highly variable situations, we gain some confidence that the inputs will hold, although we do not know whether other values would also work. We do not claim that our model finds the true values for any of the inputs, which may not in fact represent knowable quantities. Instead, we focus on developing a model that will provide useful insights about the dynamics and inter-relationships of the various components, which we hope will provide a good basis for then considering the implications of potential changes. Thus, we build the model to develop a reference case that reasonably represents our understanding of reality and we require consistency with any available data. We primarily focused on fitting the dynamics of the time series of the multiple types of incidence data (including the zero values that correspond to a lack of observed cVDPVs) and die out, and we placed secondary weight on the reported estimates of zero-dose children reported in the NPAFP data. We then hold all of the model inputs from the reference case constant and explore the implications of changing the targeted age ranges. In this context, any errors should apply equally for all scenarios considered so long as we make comparisons to the reference case. For each setting we face significant limitations related to the quality of the field and epidemiological data. For example, no information exists about the actual true vaccine coverage achieved for any round, the probability of repeatedly missing or reaching children with SIAs, or the take rates achieved for each serotype of vaccine. We also created the concept of somewhat isolated, under-vaccinated subpopulations to account for some of the heterogeneity that exists in the population and the need to capture this in the context of a differential equation based model. Unlike simple models, which might focus on fitting values of one or more of a small number of inputs for the purpose of finding the best-fit value of the inputs, our models focus on the interdependent system and finding inputs that provide reasonable agreement to all of the existing data. In this context, independently varying individual model inputs (e.g., R_0_) in a sensitivity analysis would not provide meaningful results because these different values may no longer produce a reasonable representation of the historical experience. For example, with a lower R_0_ and all else equal, WPV2 and WPV3 would die out much earlier than observed, while with higher R_0_, WPV2 may persist for much longer than observed.

**A4. Further details of reference cases**

We updated a number of model inputs for the scenarios considered based on a more appropriate SIA characterization and access to additional country-specific data (e.g., NPAFP data).[[1](#_ENREF_1)] Most changes involve relatively small variations within wide uncertainty ranges for inputs for which no direct measurements exist (Table 1 in the main paper).

For Tajikistan, the last large-scale SIAs prior to the 2010 outbreak date from 8 years before, which implies relatively little impact of these SIAs on the 2010 outbreak. In the absence of data on the quality of early SIAs in Tajikistan or large enough numbers of NPAFPs cases to reliably estimated zero-dose proportions over time, we assume true coverage of 0.8 for each pre-outbreak SIA, consistent with typical relatively good SIA quality in former Soviet Republics. Given the existence of hard-to-reach or underserved groups in Tajikistan, we assume repeated miss probabilities of 0.7 for each SIA. For the response to the 2010 outbreak, data indicate very high coverage in each round.[[10](#_ENREF_10)] At the time of the first outbreak response SIA, virtually all children targeted represent newly born children since the last previous SIAs, so the choice of *P_RM_* leads to almost no impact on the model results (i.e., all targeted children remain subject to *TC*). However, for subsequent response rounds we assume a relatively high risk of again getting missed for the small fraction that did not receive a dose in the prior round (i.e., *P_RM_* = 0.8). These assumptions lead to a slight change in the model result compared to the previously published SIA characterization,^[^[^1^](#_ENREF_1)^]^ which led us to adjust the assumption about over-reporting of routine immunization coverage due to unregistered children from 0.9 to 0.88 in order to again obtain a modeled outbreak consistent with the reported data.

For northwestern Nigeria we now assume that DTP coverage rates in existing surveys provide a more accurate measurement of true routine polio immunization coverage than POL,[[11](#_ENREF_11)] which we previously used.^[^[^1^](#_ENREF_1)^]^ We also determined that with the missed children implied by the updated SIA characterization kept close to the NPAFP data, a slightly lower R_0_ provides more realistic incidence during the years affected by the suspension of vaccination in some northern Nigerian states (2003-6).[[11](#_ENREF_11)] We assumed for simplicity that the chronically under-vaccinated subpopulation receives no routine polio vaccinations at all (instead of a very low routine immunization coverage level), adjusted the seasonality for R_0,_ and modified some of the take rates. For India, access to the AFP data dating back to 1997 revealed that we previously assumed many more missed children than supported by the NPAFP data. This led to more significant changes, including characterization of large under-vaccinated subpopulations in both Bihar and Western Uttar Pradesh to sustain transmission through 2011 despite extremely low zero-dose proportions among NPAFP cases in the general population. In addition to these changes, Figures A3-A5 show the assumed true coverage and repeated miss probabilities for each SIA in the three situations. For northwestern Nigeria and northern India, we characterized the SIAs to produce implied zero-dose proportions over time among 18-month old children in the general population roughly similar to those reported over time by NPAFP cases of similar age, as shown in Figures A6 and A7. Figures A8-A10 show the updated model results compared to the available data and the results reported by Duintjer Tebbens et al. (2013).[[1](#_ENREF_1)] Small discrepancies between the kinetics of the model and reported data arise because the differential-equation based model does not account for stochasticity (e.g., tracking who becomes a paralytic case or not if infected for the first time, or the die-out of viruses) and the real heterogeneity that exists in the populations (i.e., we do not model how the virus moves from one community to another, but focus on the overall dynamics and population immunity).[[1](#_ENREF_1)] Larger discrepancies (e.g., in northwest Nigeria, and in northern India before the early 2000s) suggest that surveillance probably seriously underestimated the true incidence, which remains consistent with our understanding of surveillance quality in the different situations over time. In some cases (e.g., type 2 in northwest Nigeria in 2009), our simplification of the heterogeneity also probably contributes to larger discrepancies.[[1](#_ENREF_1)] In northern India, very intense surveillance in recent years may imply that some children with AFP did not experience paralysis due to poliovirus despite poliovirus isolation, particularly during outbreak peaks that coincide with high WPV prevalence in the populations. We expect the most overestimation of cases by surveillance for WPV3 and cVDPV2 because of their low paralysis-to-infection ratios which imply a high total prevalence of WPV per true paralytic case. Moreover, the paralysis-to-infection ratios used in our model reflect rates of residual paralysis,[[12](#_ENREF_12)]while AFP surveillance also classifies cases of AFP without residual paralysis (but with WPV isolated) as WPV-confirmed polio.

**Figure A1: Waning curves for fecal-oral and oropharyngeal transmission for individuals with 1 prior LPV infection or 2 or more prior LPV infections.**

**
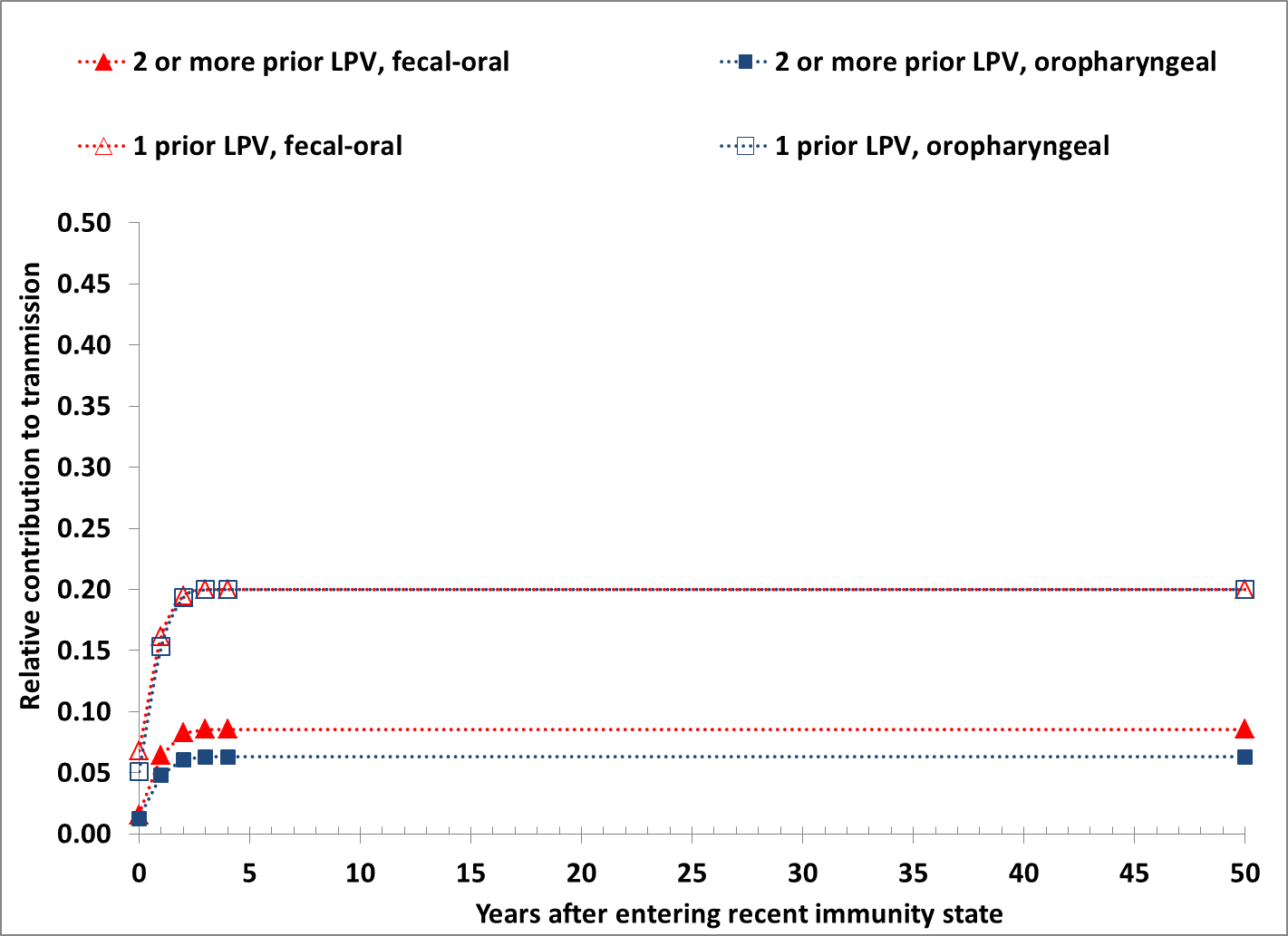
**

**Figure A2: SIA tree showing the probabilities of receiving a dose in two subsequent rounds**

**Figure A3: Assumed characterization of SIAs in Tajikistan.**

**(a) True coverage (*TC*)**

**
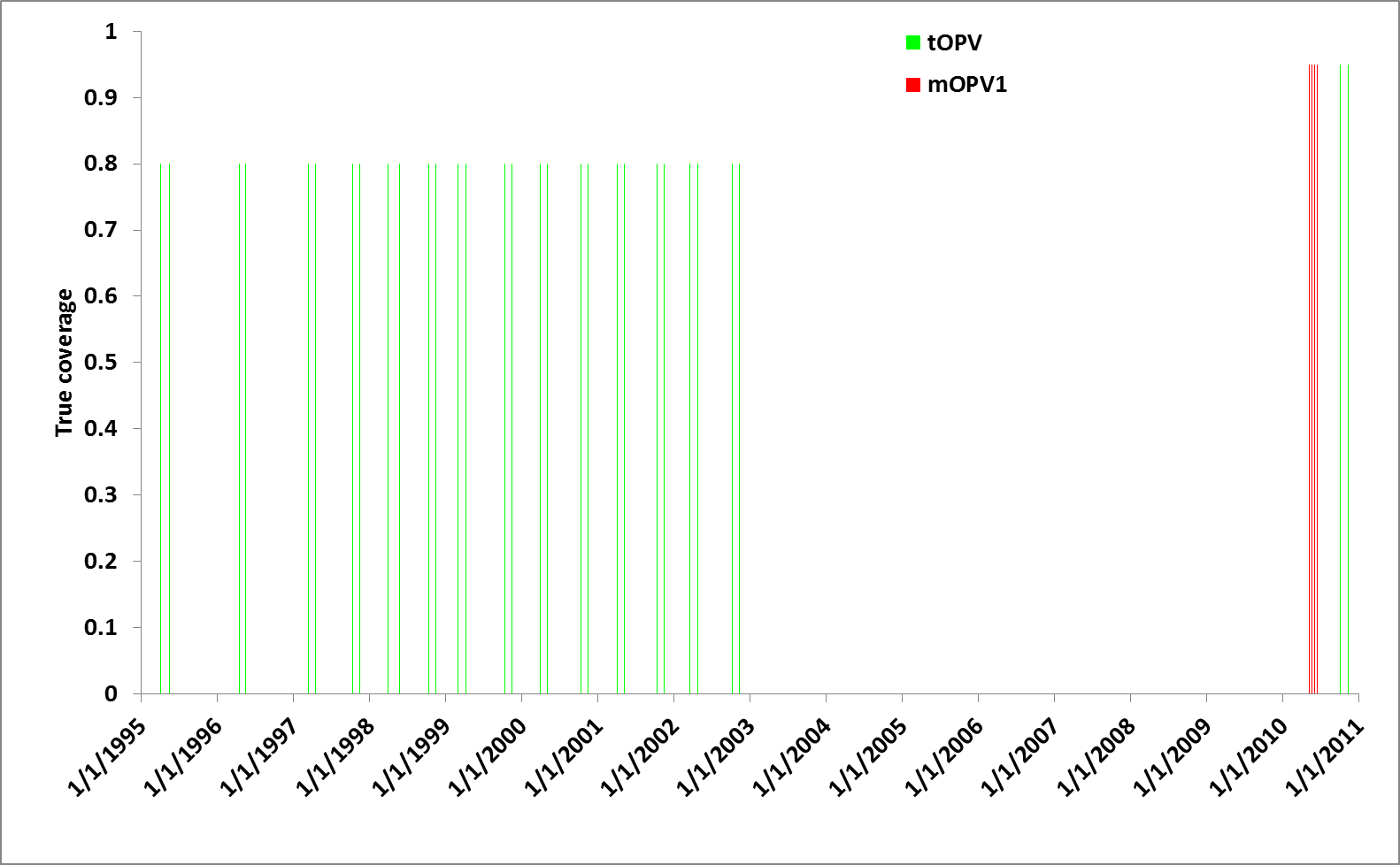
**

**(b) Repeated missed probability (*P_RM_*)**

**
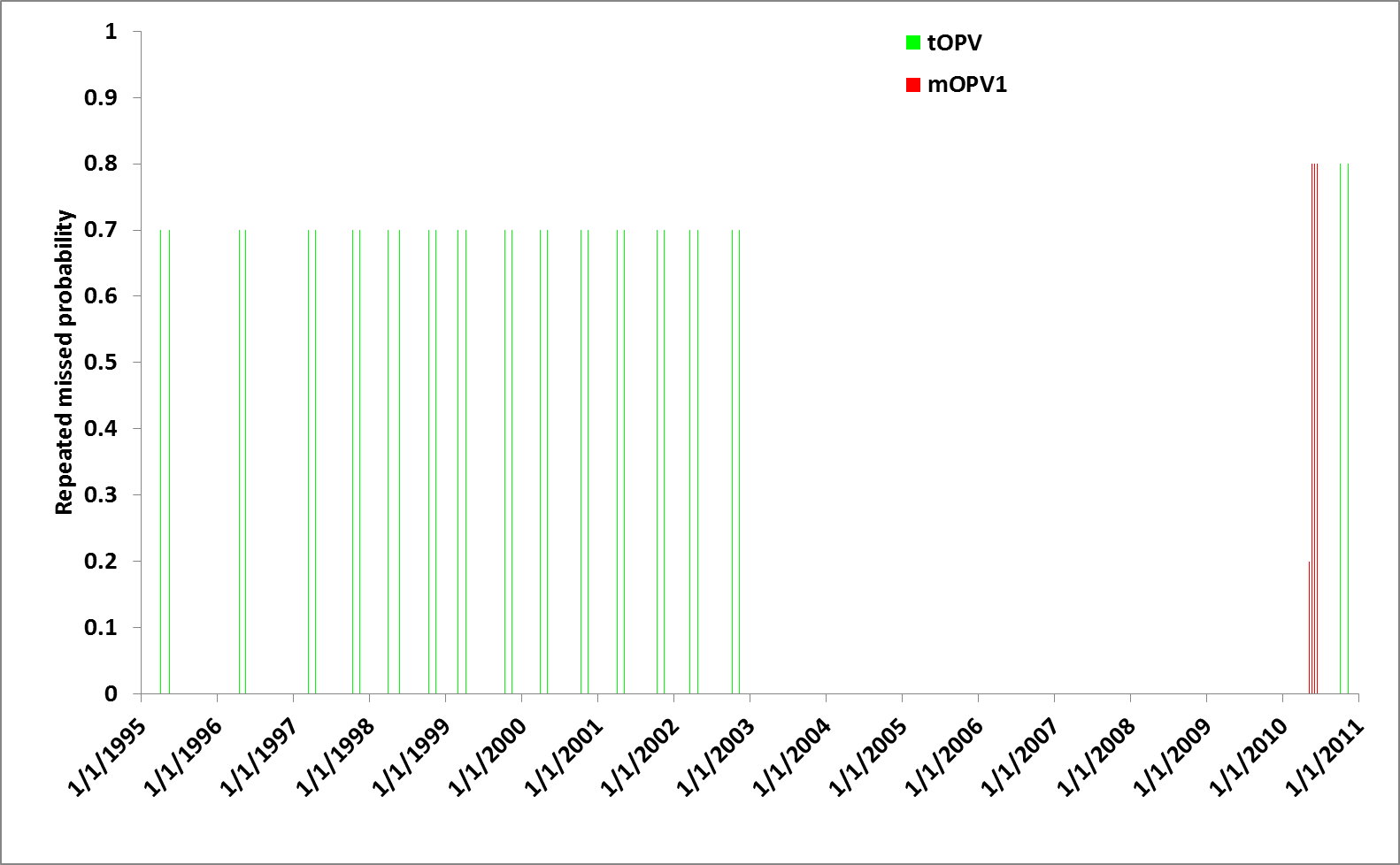
**

**Figure A4: Assumed characterization of SIAs in northern India.**

**(a) True coverage (*TC*) times fraction targeted (*F*), general population, Bihar**

**
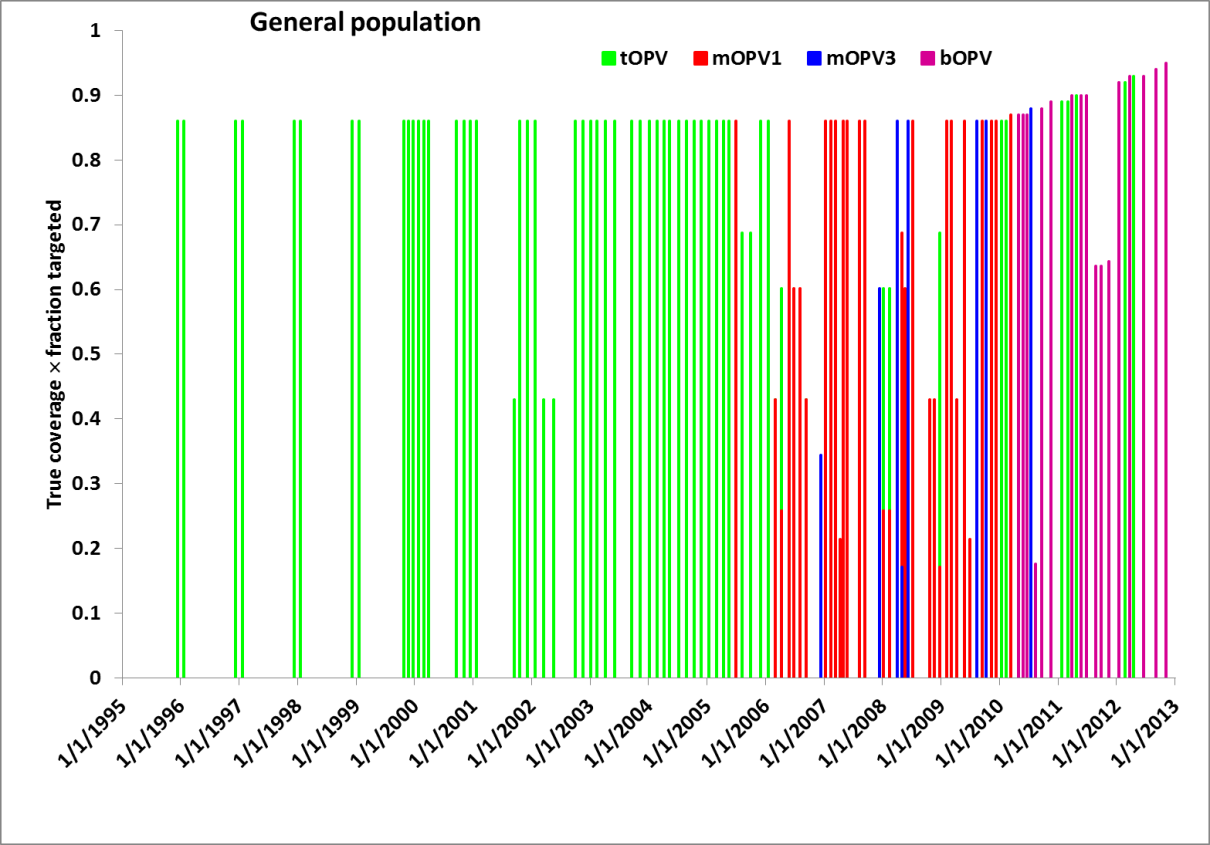
**

**(b) True coverage (*TC*) times fraction targeted (*F*), under-vaccinated subpopulation, Bihar**

**
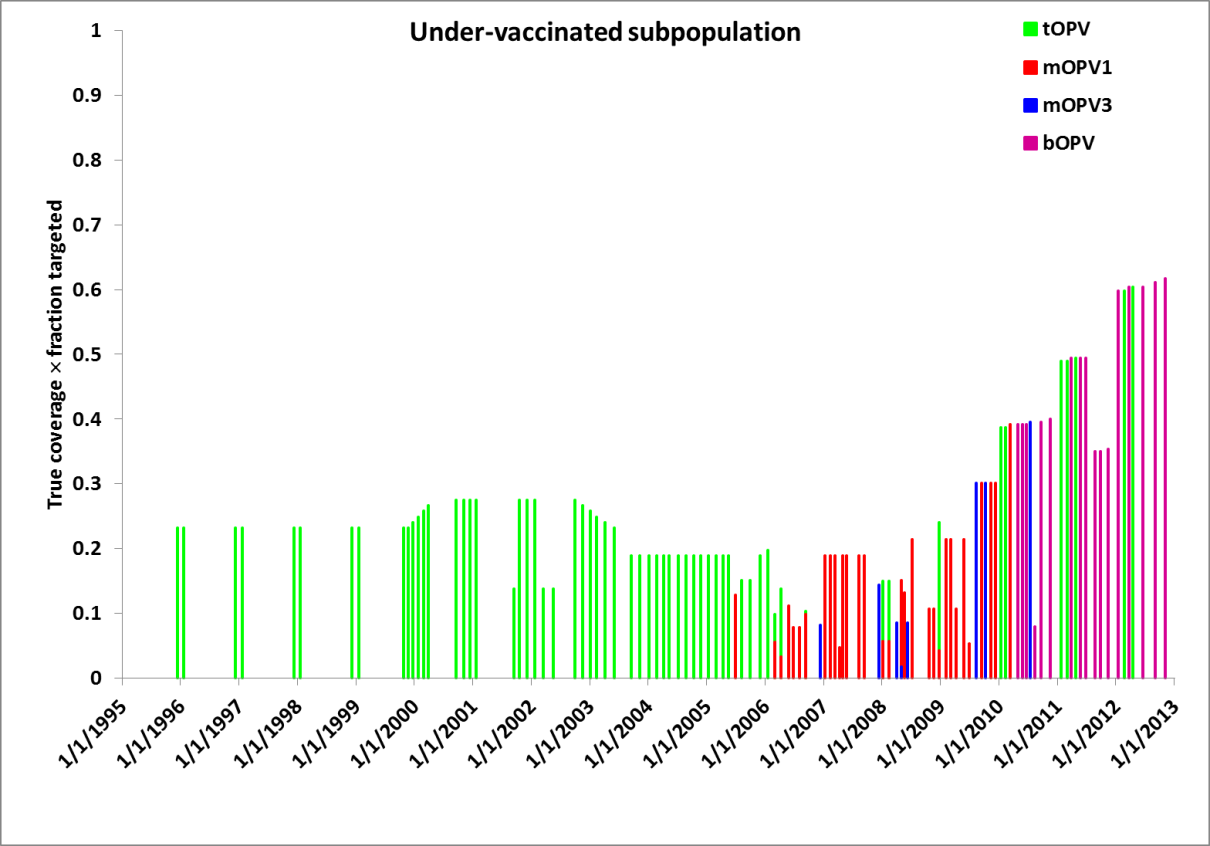
**

**(c) True coverage (*TC*) times fraction targeted (*F*), general population, WUP**

**
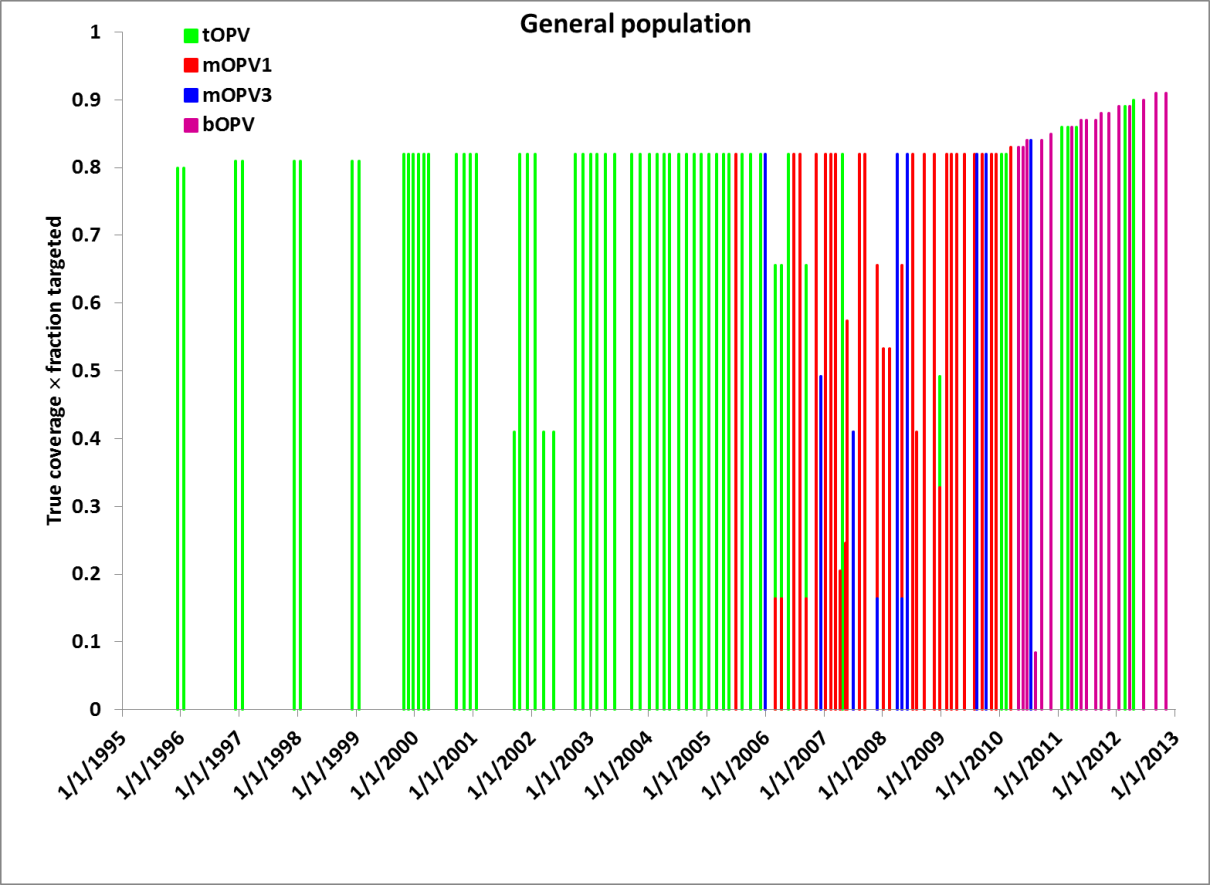
**

**(d) True coverage (*TC*) times fraction targeted (*F*), under-vaccinated subpopulation, WUP**

**
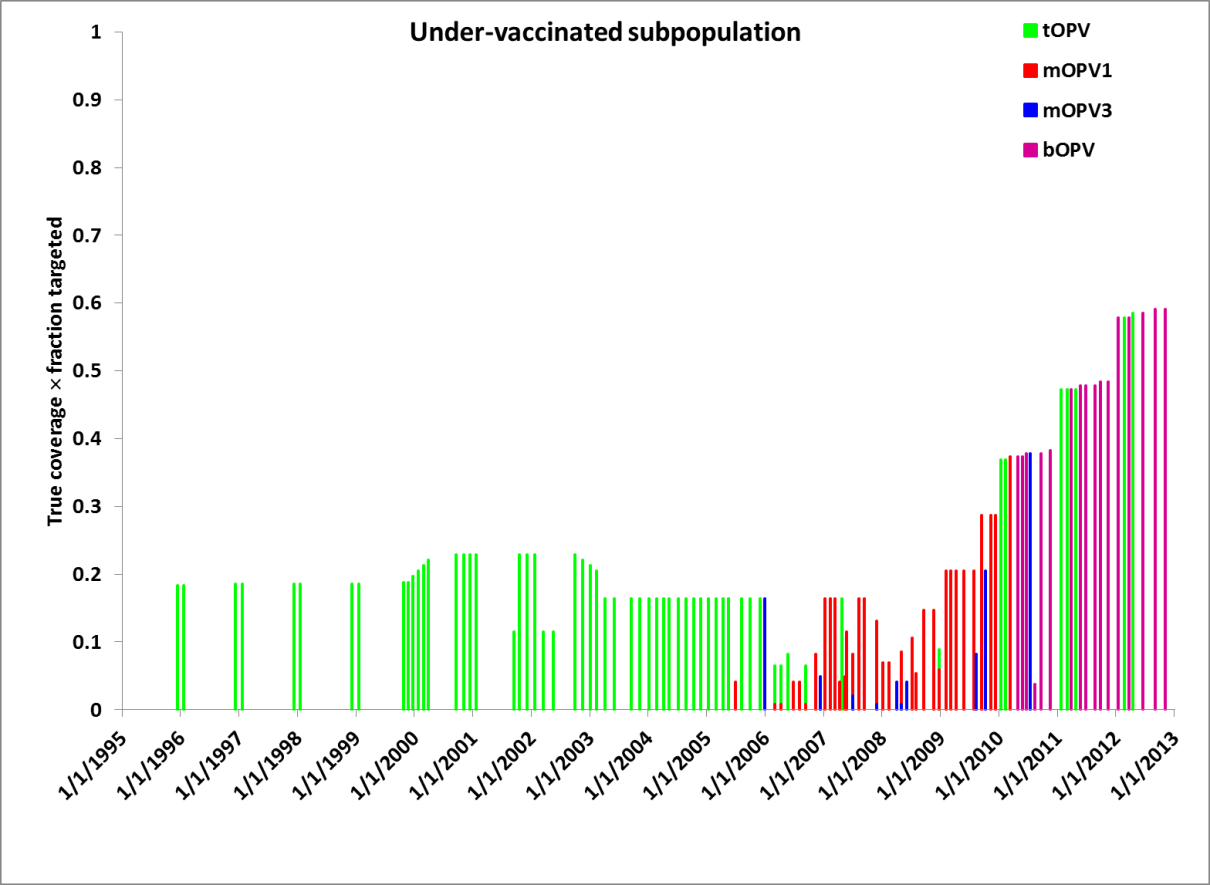
**

**(e) Repeated missed probability (*P_RM_*), general population, Bihar**

**
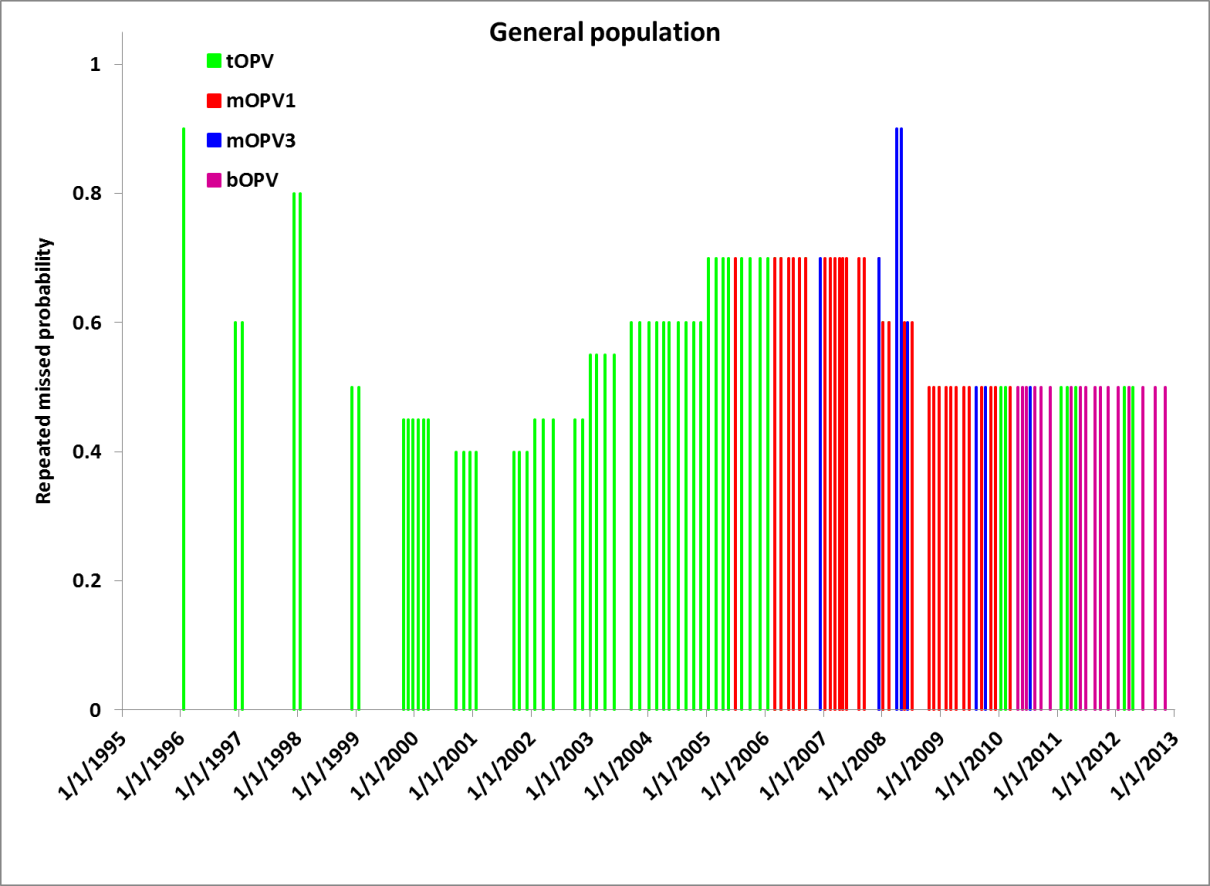
**

**(f) Repeated missed probability (*P_RM_*), under-vaccinated subpopulation, Bihar**

**
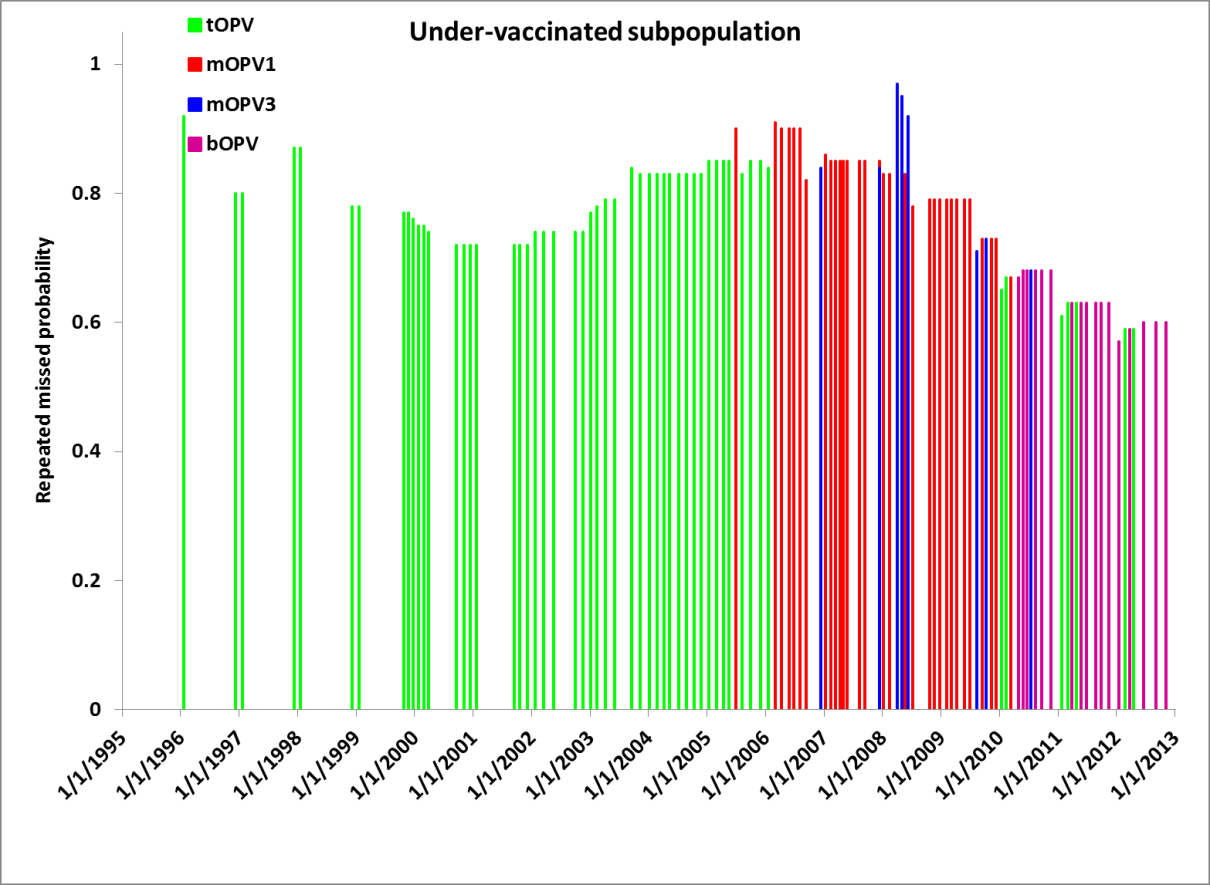
**

**(g) Repeated missed probability (*P_RM_*), general population, WUP**

**
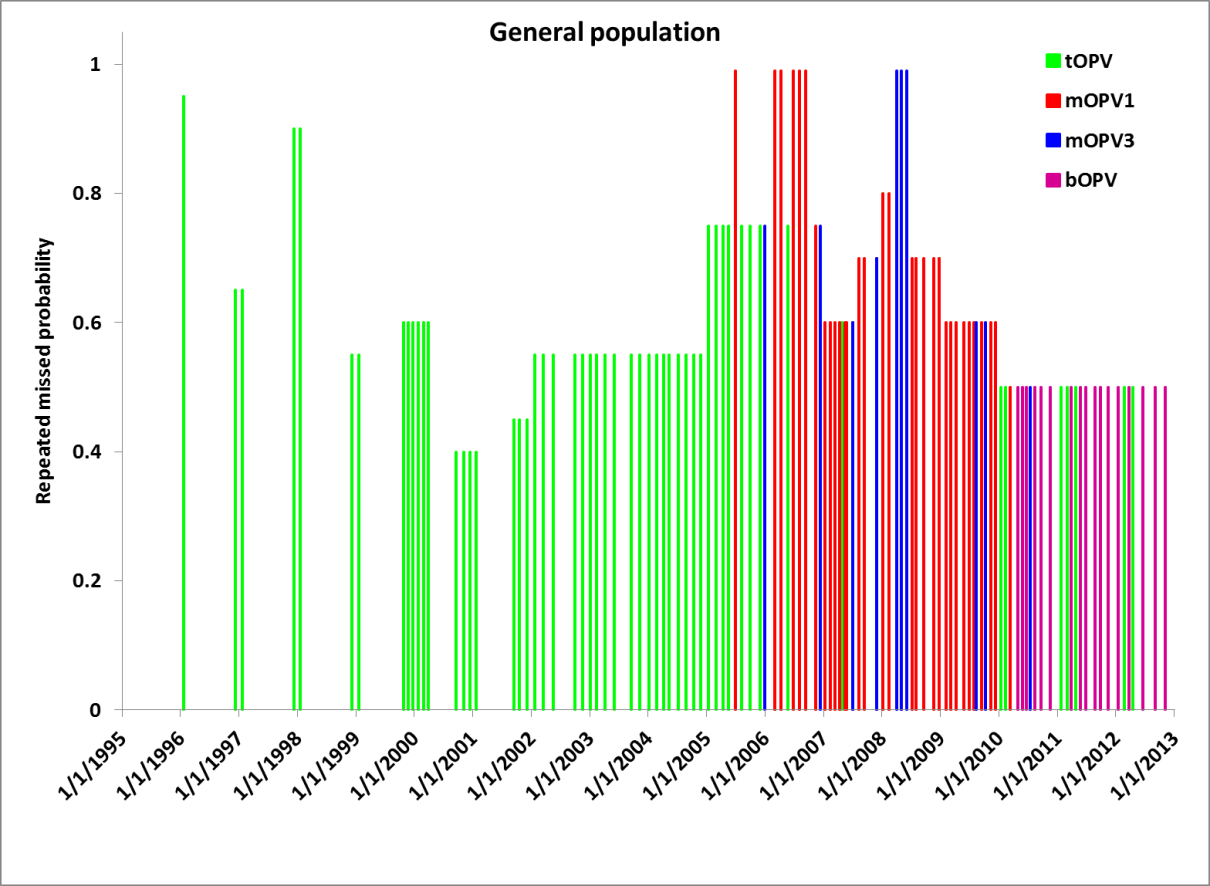
**

**(h) Repeated missed probability (*P_RM_*), under-vaccinated subpopulation, WUP**

**
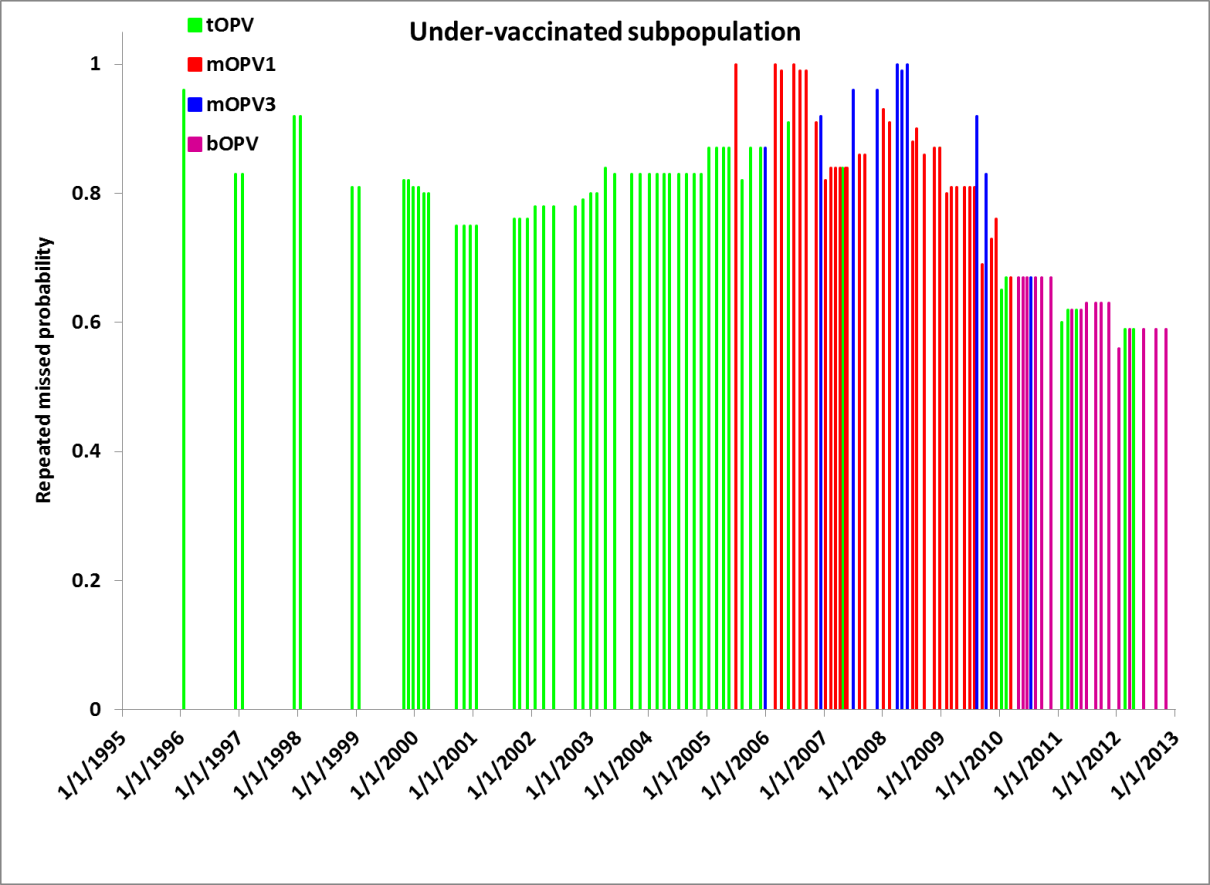
**

**Figure A5: Assumed characterization of SIAs in northern Nigeria.**

**(a) True coverage (*TC*) times fraction targeted (*F*), general population**

**
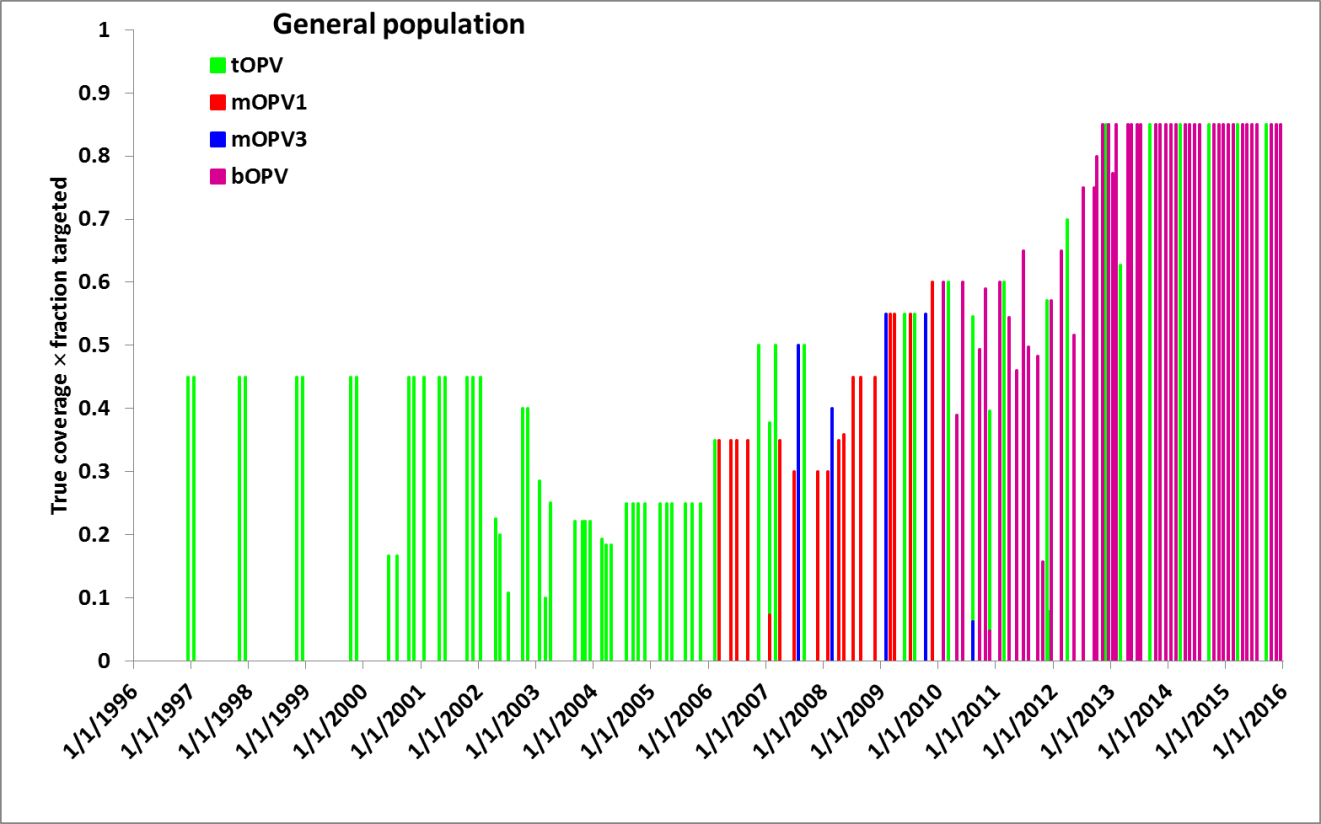
**

**(b) True coverage (*TC*) times fraction targeted (*F*), under-vaccinated subpopulation**

**
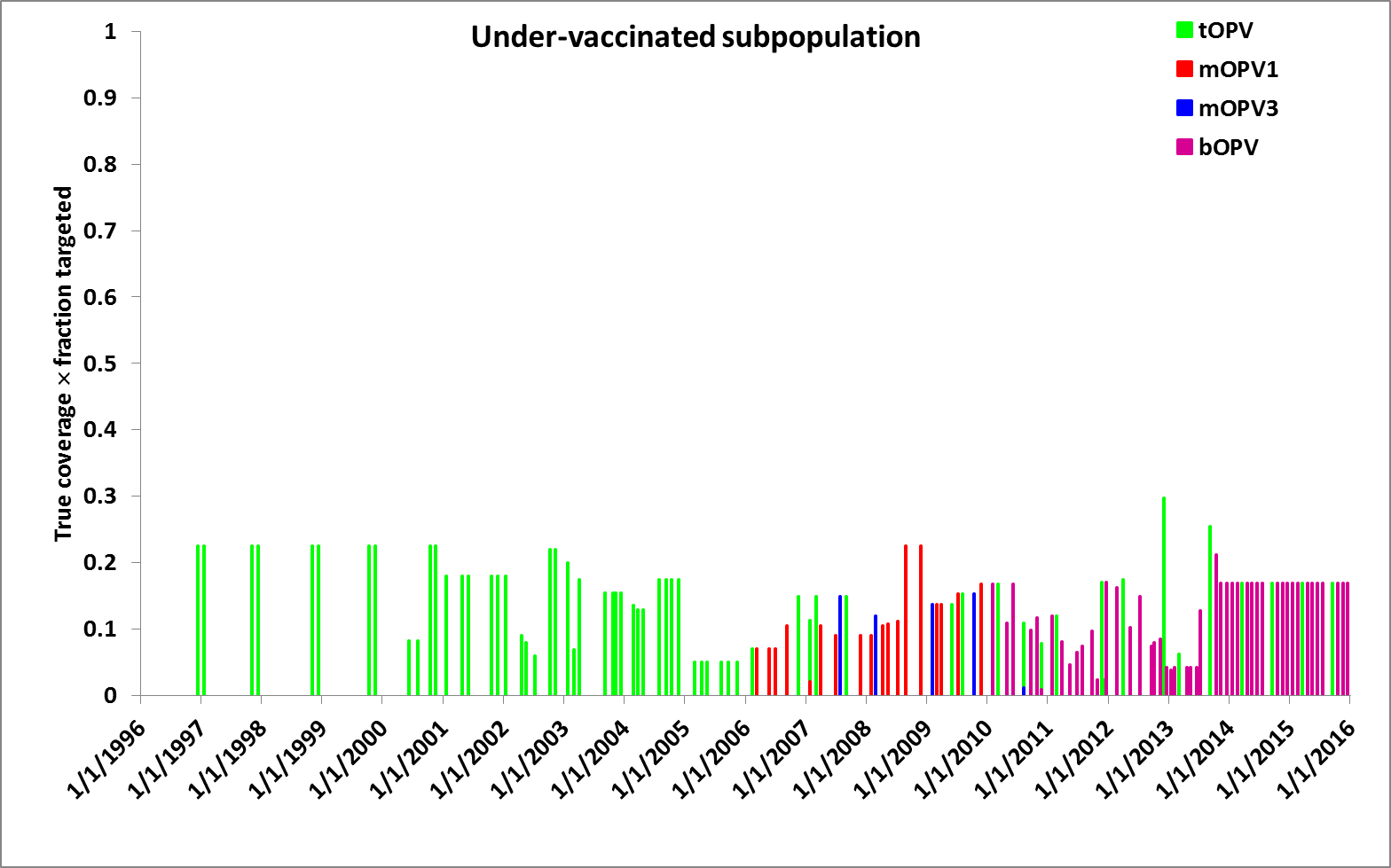
**

**(c) Repeated missed probability (*P_RM_*), general population**

**
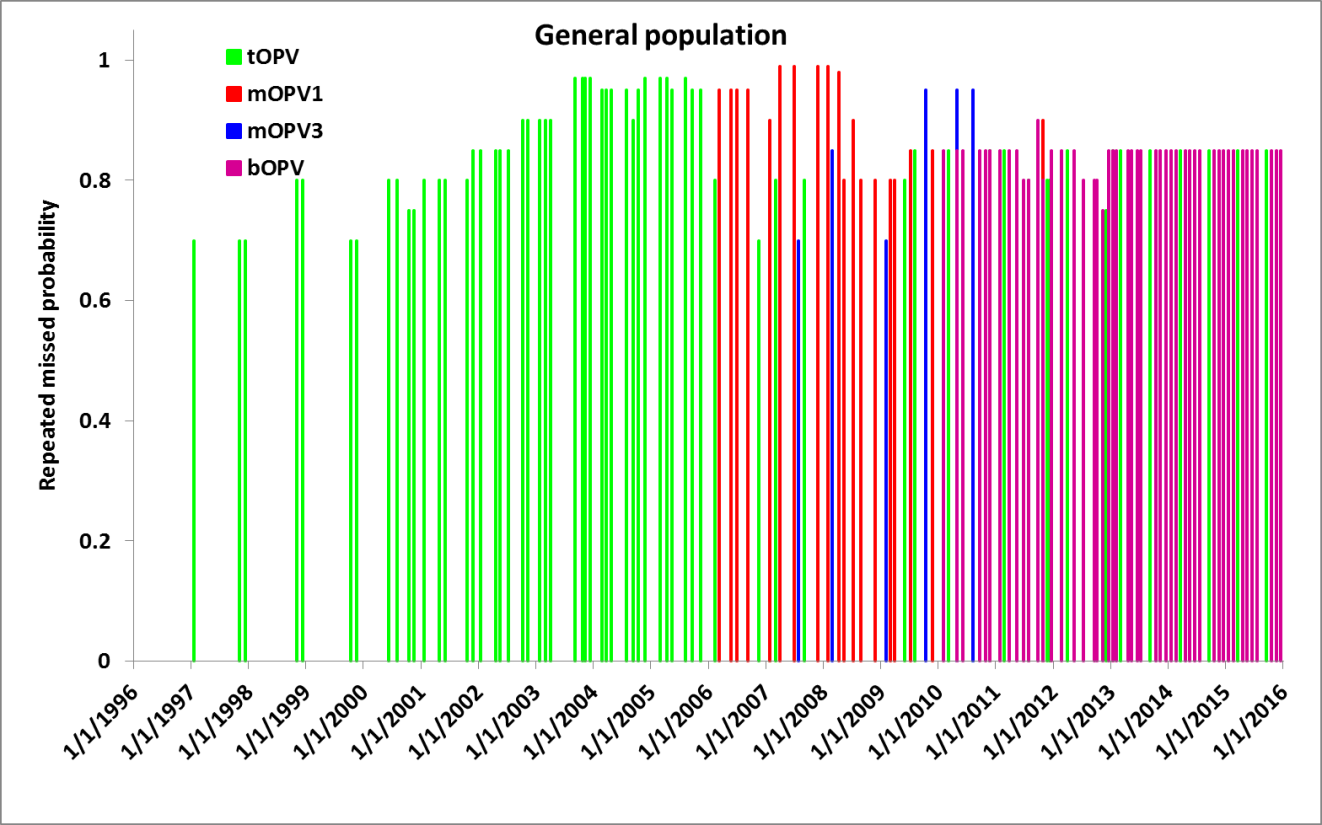
**

**(d) Repeated missed probability (*P_RM_*), under-vaccinated subpopulation**

**
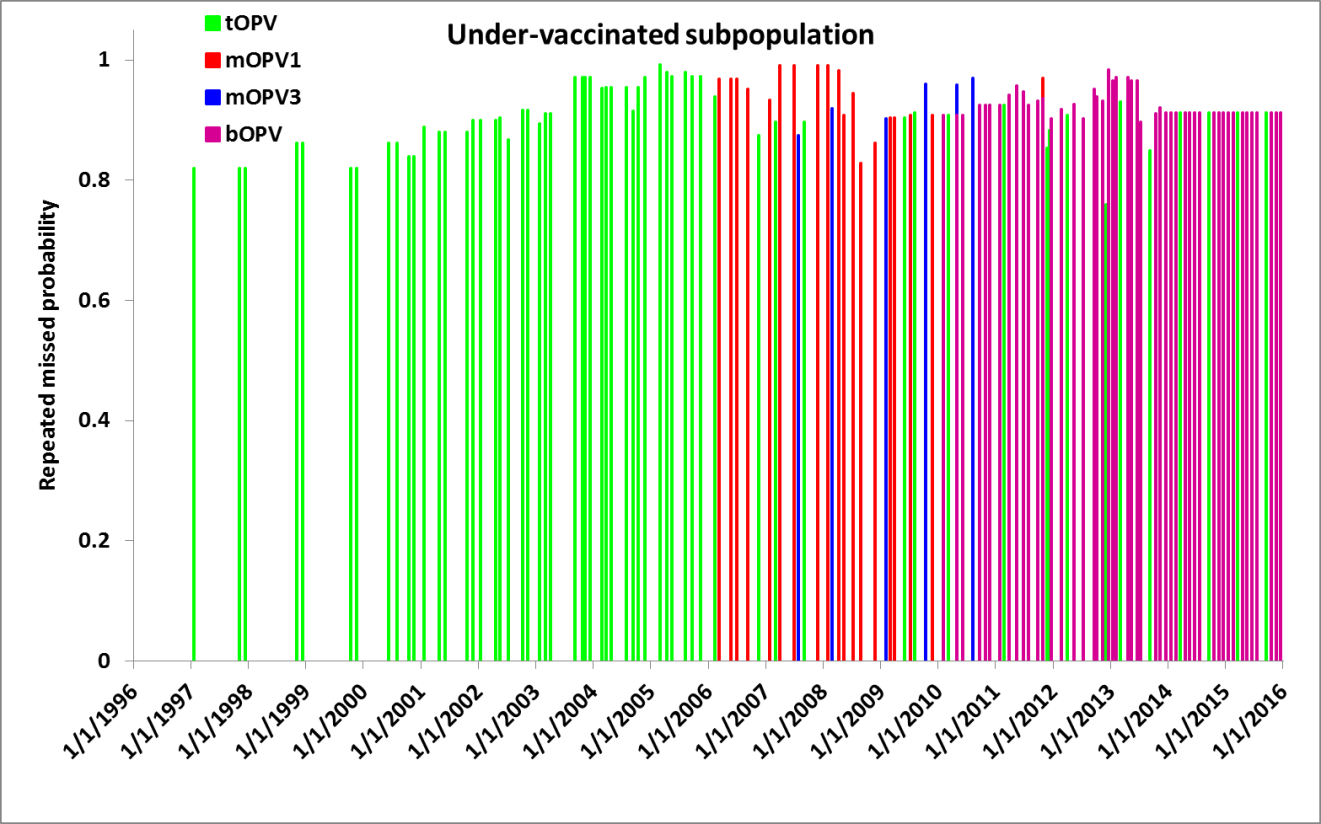
**

**Figure A6: Zero-dose proportions implied by the assumed characterization of SIAs in northern India.**

**(a) Bihar**

**
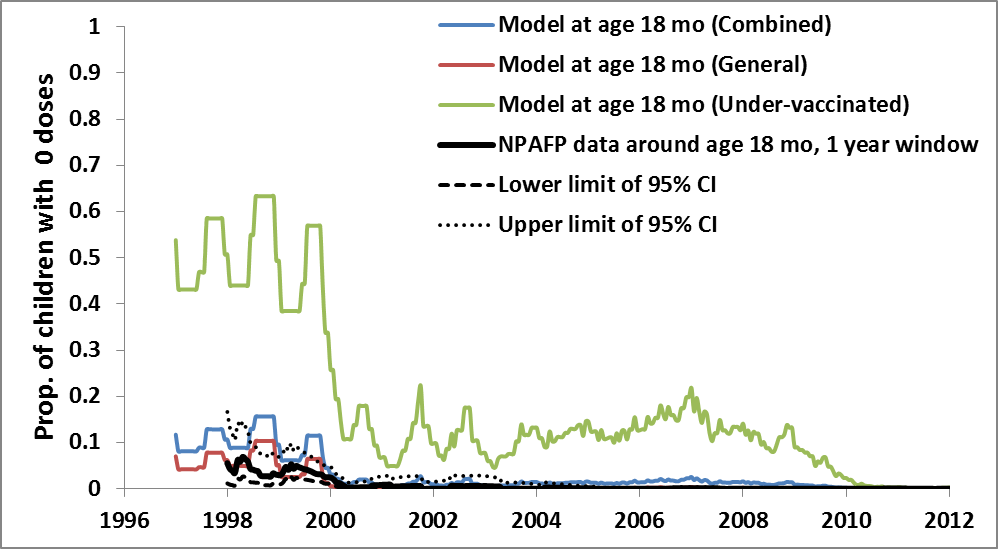
**

**(b) Western Uttar Pradesh**

**Figure A7: Zero-dose proportions implied by the assumed characterization of SIAs in northern Nigeria.**

**
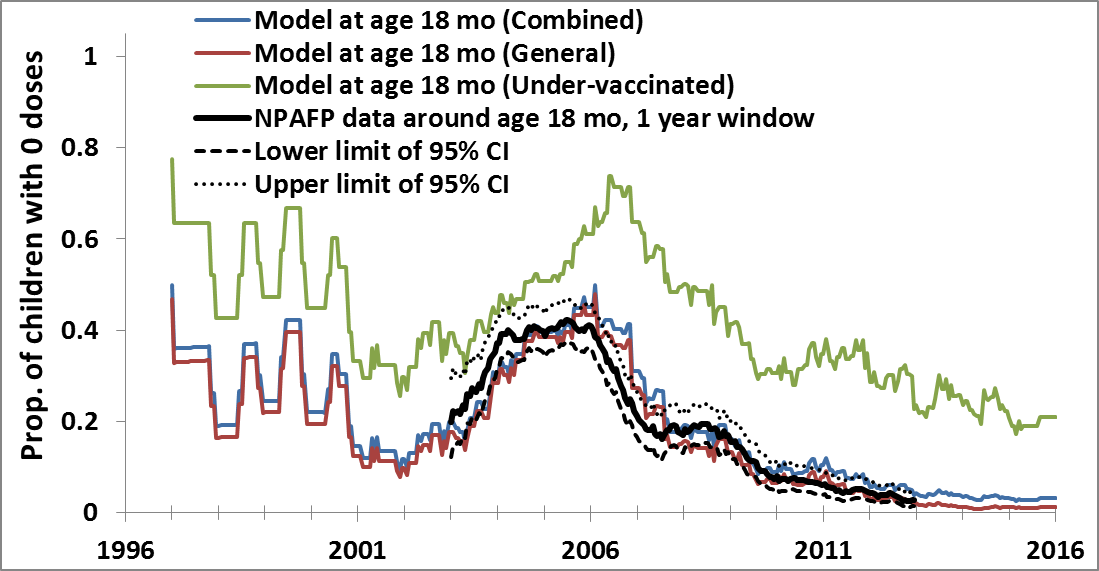
**

**Figure A8: Model for Tajikistan, showing the updated result and the result from Duintjer Tebbens et al. (2013).[**[**1**](#_ENREF_1)**]**

**
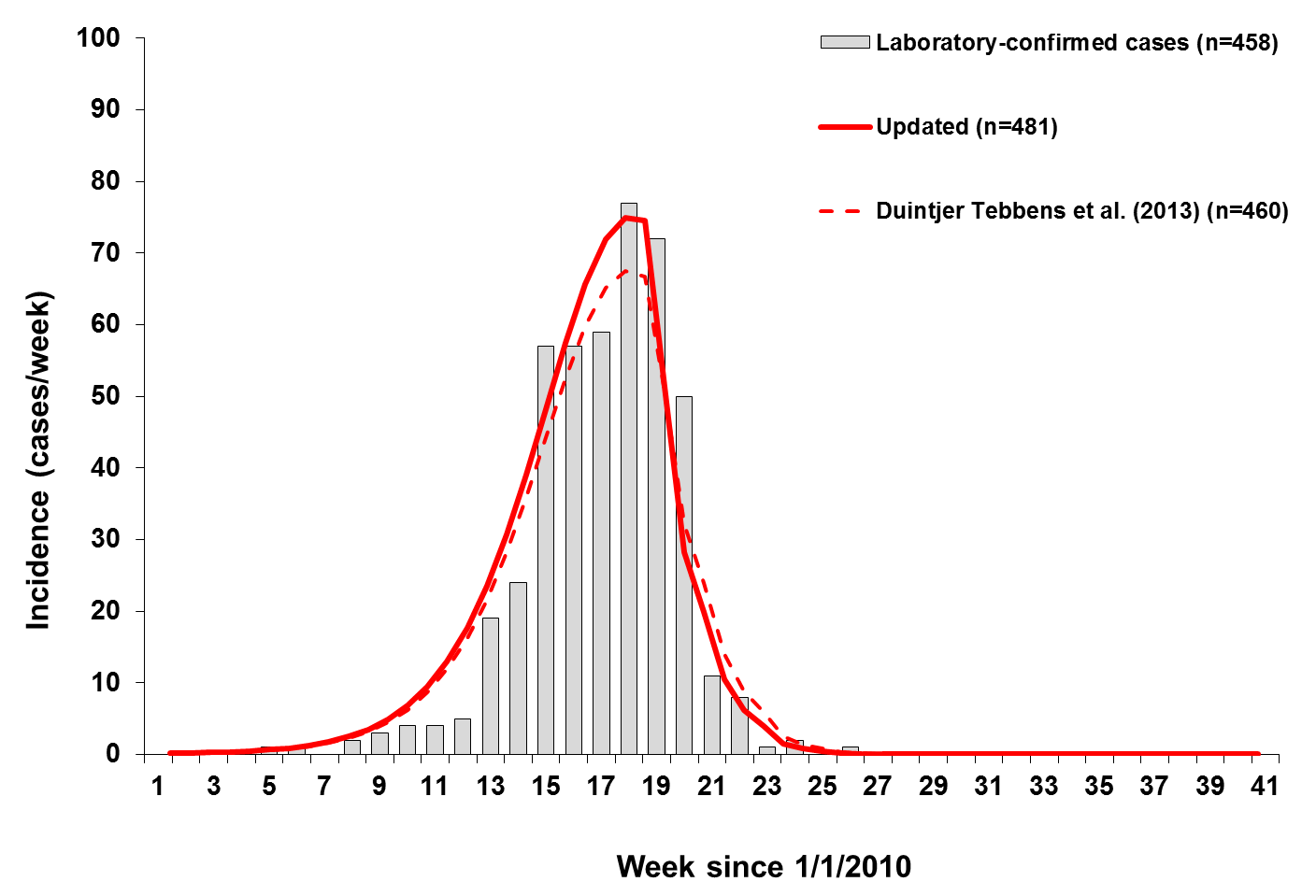
**

**Figure A9: Model for northern India, showing the updated result and the result from Duintjer Tebbens et al. (2013).[**[**1**](#_ENREF_1)**]**

**(a) Type 1**

**
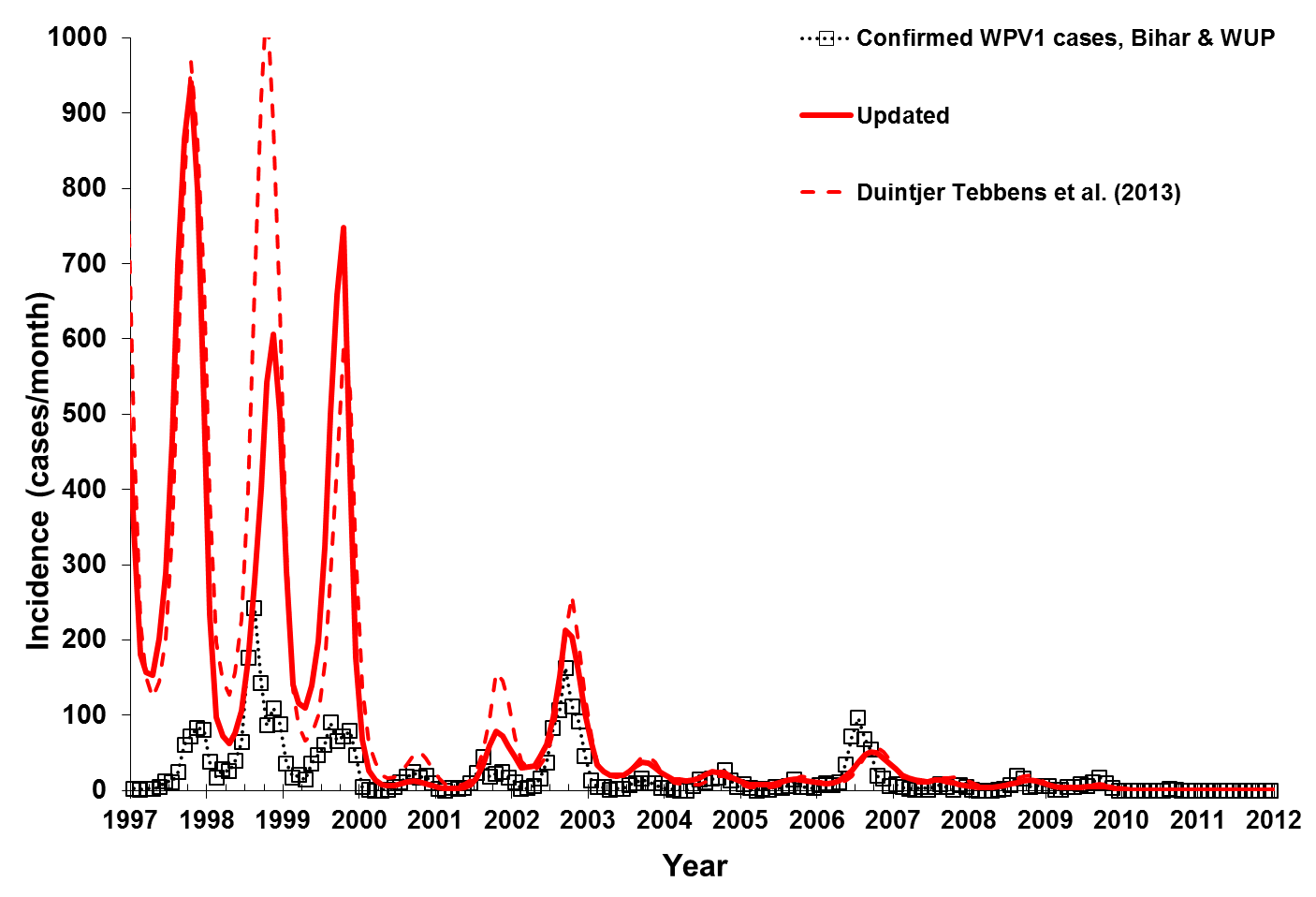
**

**(b) Type 2 (model incidence includes only last reversion stage corresponding to cVDPVs)**

**
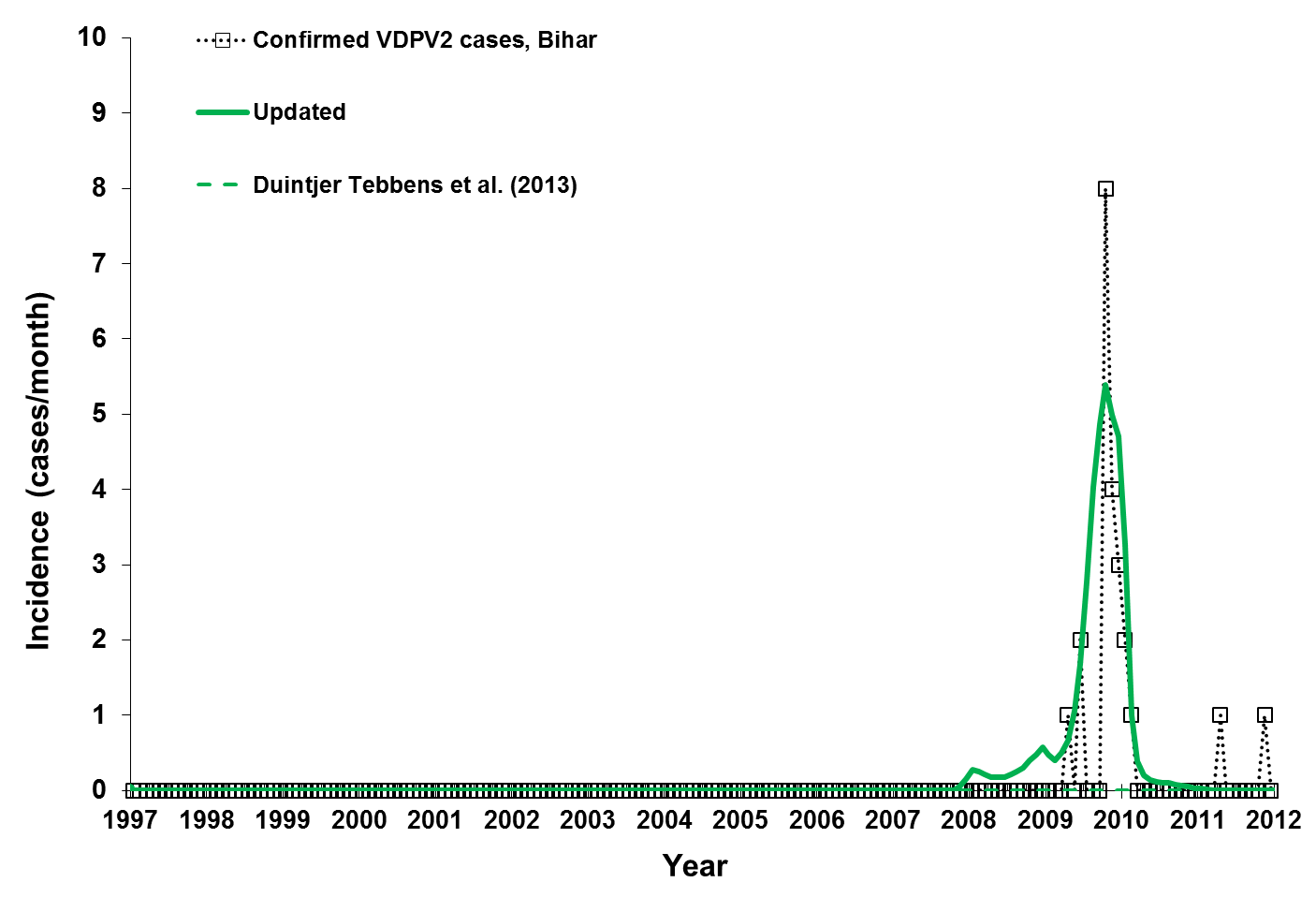
**

**(c) Type 3**

**
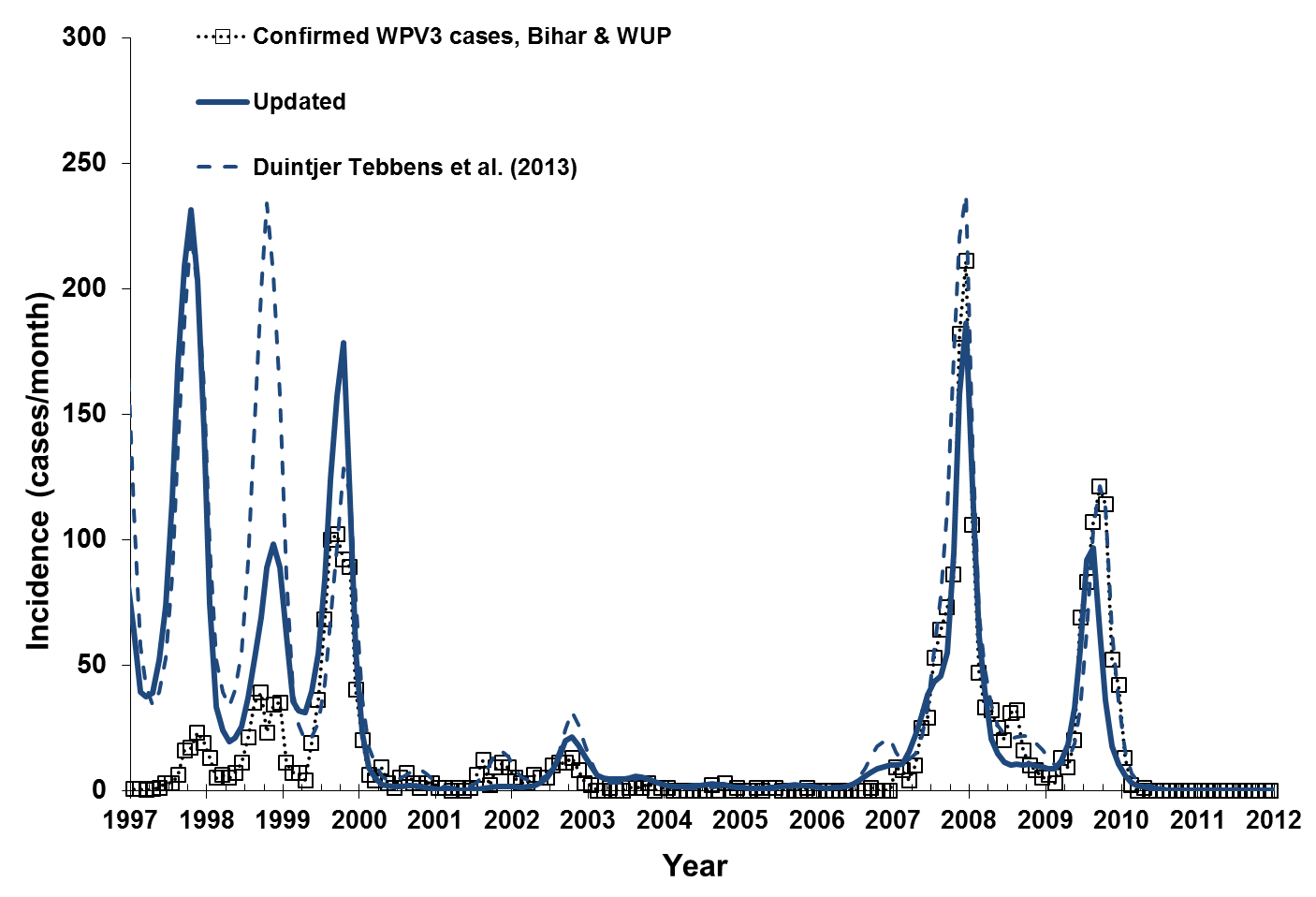
**

**Figure A10: Model for northwestern Nigeria, showing the updated result and the result from Duintjer Tebbens et al. (2013).[**[**1**](#_ENREF_1)**]**

**(a) Type 1**

**
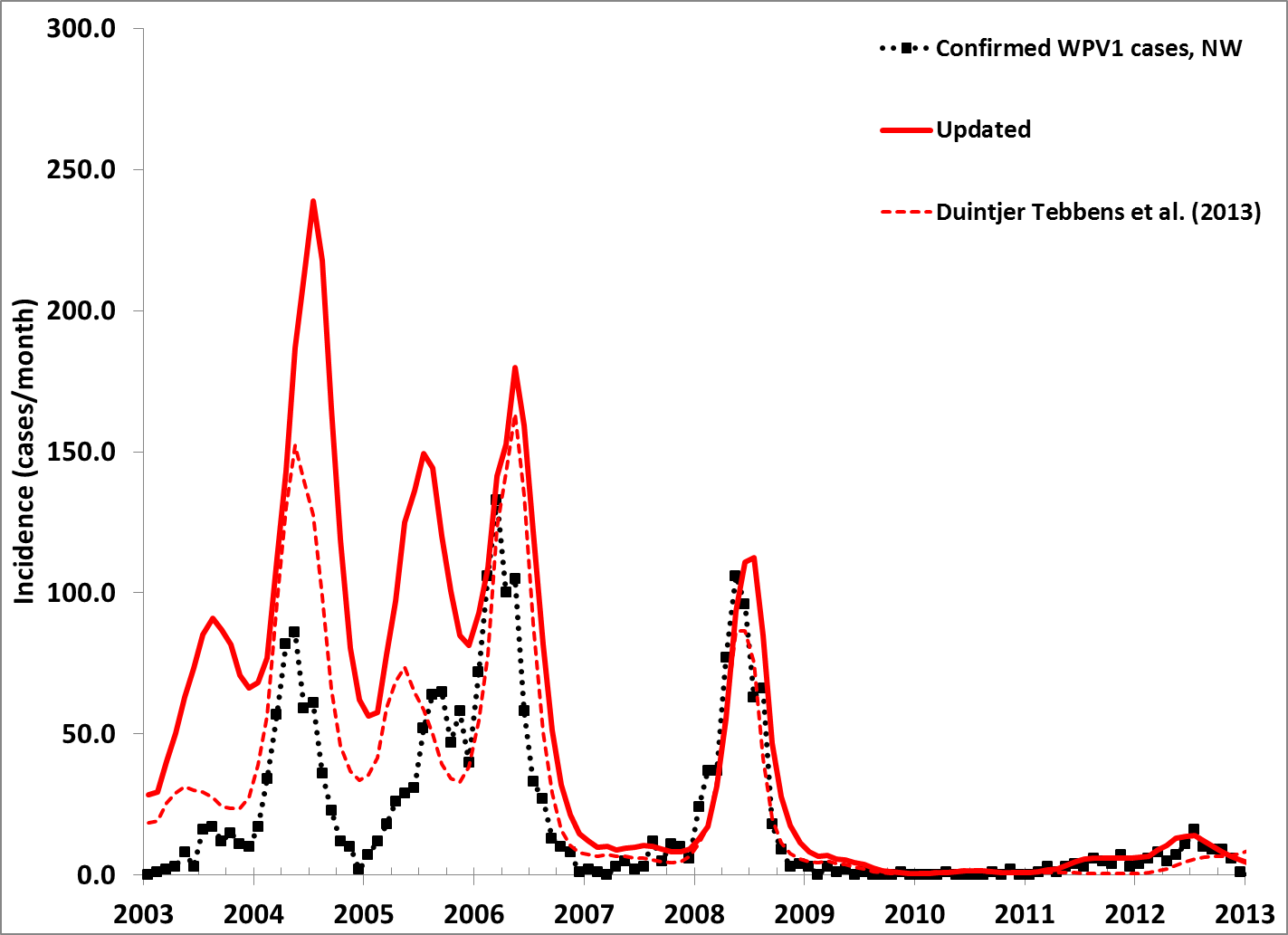
**

**(b) Type 2 (model incidence includes only last reversion stage corresponding to cVDPVs)**

**
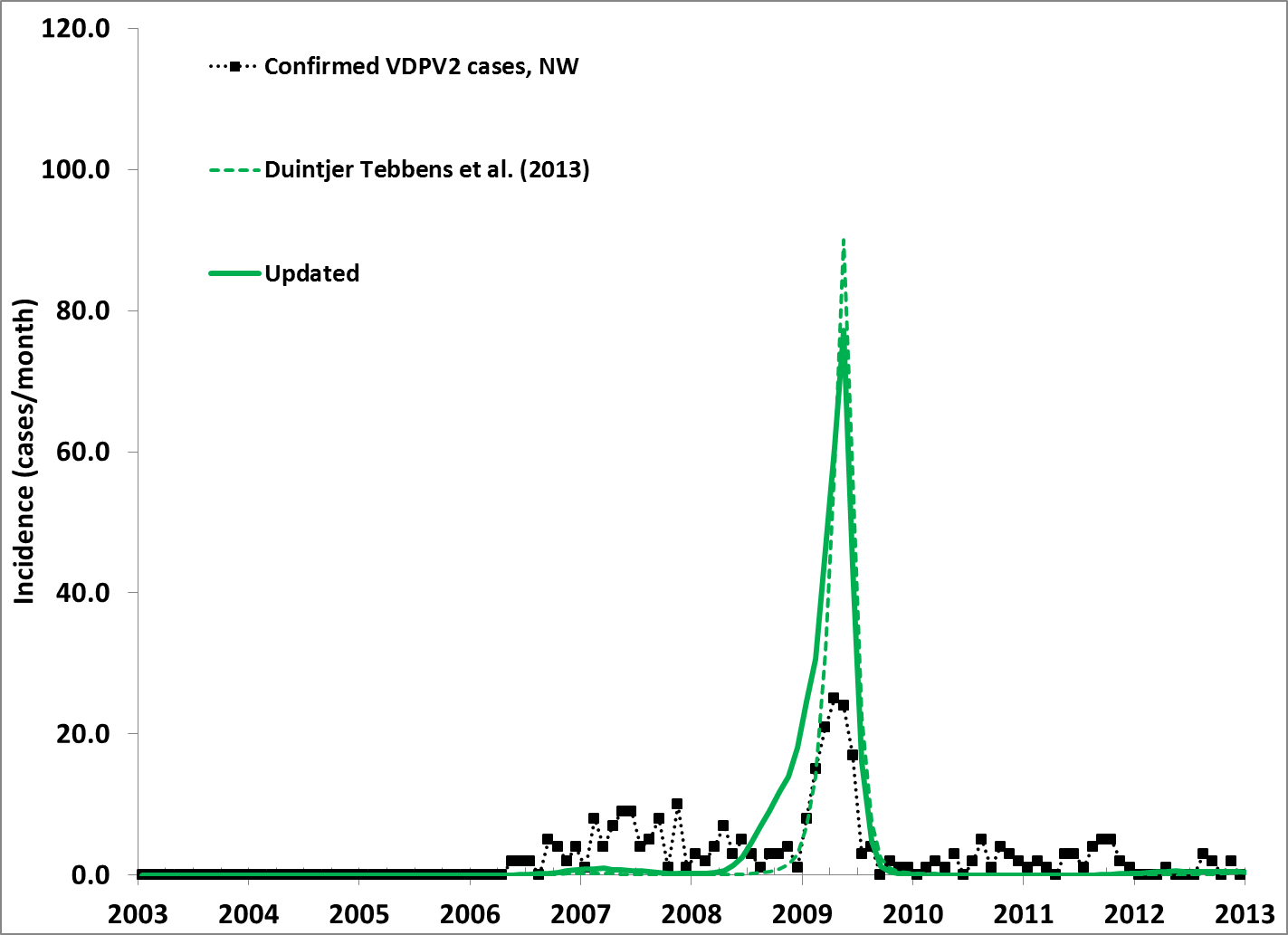
**

**(c) Type 3**

**
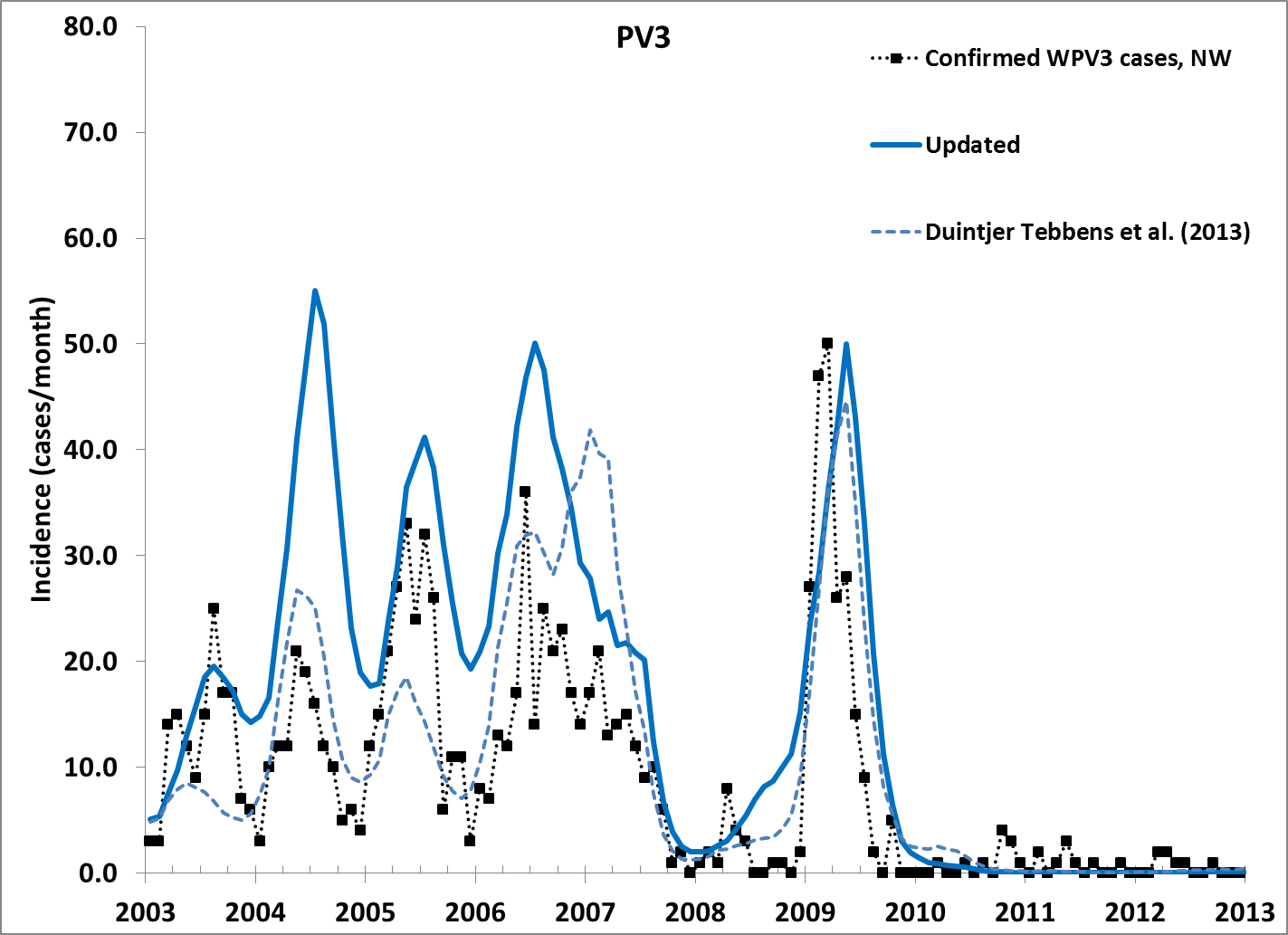
**

**References:**

1. Duintjer Tebbens RJ, Pallansch MA, Kalkowska DA, Wassilak SG, Cochi SL, Thompson KM: **Characterizing poliovirus transmission and evolution: Insights from modeling experiences with wild and vaccine-related polioviruses.** *Risk Anal* 2013, **23**:703-749.

2. Duintjer Tebbens RJ, Pallansch MA, Kew OM, Cáceres VM, Sutter RW, Thompson KM: **A dynamic model of poliomyelitis outbreaks: Learning from the past to help inform the future.** *Am J Epidemiol* 2005, **162**:358-372.

3. Glasser J, Feng Z, Moylan A, Del Valle S, Castillo-Chavez C: **Mixing in age-structured population models of infectious diseases.** *Math Biosci* 2012, **235**:1-7.

4. Jacquez JA, Simon CP, Koopman J, Sattenspiel L, Perry T: **Modeling and analyzing HIV transmission: the effect of contact patterns.** *Math Biosci* 1988, **92**:119-199.

5. Thompson KM, Pallansch MA, Duintjer Tebbens RJ, Wassilak SG, Kim J-H, Cochi SL: **Pre-eradication vaccine policy options for poliovirus infection and disease control.** *Risk Anal* 2013, **33**:516-543.

6. Demographic and Health Surveys, Nigeria 1990, 1999, 2003, and 2008 http://measuredhs.com/

7. Demographic and Health Surveys, India, 1992-93, 1998-99, 2005-06

http://measuredhs.com/

8. State Committee on Statistics of the Repubic of Tajikistan: Tajikistan Living Standards Measurement Survey 2007. State Committee on Statistics of the Republic of Tajikistan, Dushanbe, Tajikistan; 2009.

9. Linear Dependence between Two Bernoulli Random Variables http://demonstrations.wolfram.com/LinearDependenceBetweenTwoBernoulliRandomVariables/

10. Centers for Disease Control and Prevention: **Outbreaks following wild poliovirus importations --- Europe, Africa, and Asia, January 2009-September 2010.** *Morb Mortal Wkly Rep 2010*, **59**:1393-1399.

11. Wassilak SGF, Pate MA, Wannemuehler K, Jenks J, Burns C, Chenoweth P, Abanida EA, Adu F, Baba M, Gasasira A, et al: **Outbreak of type 2 vaccine-derived poliovirus in Nigeria: emergence and widespread circulation in an underimmunized population.** *J Infect Dis* 2011, **203**:898-909.

12. Nathanson N, Kew OM: **From emergence to eradication: the epidemiology of poliomyelitis deconstructed.** *Am J of Epidemiol* 2010, **172**:1213-1229.
